# Systematic review of centrifugal valving based on digital twin modeling towards highly integrated lab-on-a-disc systems

**DOI:** 10.1038/s41378-021-00317-3

**Published:** 2021-12-16

**Authors:** Jens Ducrée

**Affiliations:** School of Physical Sciences, Dublin City University, Dublin, Ireland

**Keywords:** Electrical and electronic engineering, Optical physics

## Abstract

Current, application-driven trends towards larger-scale integration (LSI) of microfluidic systems for comprehensive assay automation and multiplexing pose significant technological and economical challenges to developers. By virtue of their intrinsic capability for powerful sample preparation, centrifugal systems have attracted significant interest in academia and business since the early 1990s. This review models common, rotationally controlled valving schemes at the heart of such “Lab-on-a-Disc” (LoaD) platforms to predict critical spin rates and reliability of flow control which mainly depend on geometries, location and liquid volumes to be processed, and their experimental tolerances. In absence of larger-scale manufacturing facilities during product development, the method presented here facilitates efficient simulation tools for virtual prototyping and characterization and algorithmic design optimization according to key performance metrics. This virtual in silico approach thus significantly accelerates, de-risks and lowers costs along the critical advancement from idea, layout, fluidic testing, bioanalytical validation, and scale-up to commercial mass manufacture.

## Introduction

Since their inception in the early 1990s, an important design goal of microfluidic Lab-on-a-Chip, or micro Total Analysis Systems (µTAS)^1–5^, has been to “cram more components onto integrated circuits”, and thus provide more functionality on a given piece of (chip) real estate. This objective is somewhat on the analogy of Moore’s law^6^ that has been guiding the miniaturization of microelectronics since the 1960s. Shrinking structural dimensions is reasoned by technical aspects, e.g., functional integration for enabling modern, high-performance computers, smartphones, and gadgets, as well as economic incentives, as the cost of material and (typically pattern-transfer based) manufacturing processes strongly scales with the surface area of the chip^7^. “Price per functional unit”, and thus the packing density, may hence be deemed a paramount driver of technology development.

While the general wish lists for cost and capabilities are quite alike, microfluidics-enabled (bio-)analytical technologies can often not be downsized towards the nanoscale; this is, for instance, to still guarantee the presence of a minimum number of analyte molecules or particles in the (bio-)sample for assuring sufficient statistics, for meeting limits of detection, for avoiding drastic changes in dominant fluidic effects, such adverse surface interactions, and evaporation, along increasing surface-to-volume ratios towards miniaturization.

Over the recent decades, numerous “Lab-on-a-Chip” platforms have been developed, many of them conceived for decentralized biochemical testing^8–13^. On the one hand, these microfluidic systems may enhance the analytical performance, e.g., through expediting the completion of transport processes, such driving diffusive mixing and heat exchange for short time-to-result, by imposing highly controlled conditions under strict laminarity at low Reynolds numbers, or by scale-matching with bio-entities such as cells. On the other hand, miniaturization resides at the backbone of sample-to-answer automation and parallelization, e.g., as a crucial product requirement for deployment of bioassay panels at the point-of-use/point-of-care (PoC), and patient self-testing at home.

Lab-on-a-Chip systems frequently feature a modular setup where a microfluidic chip is inserted into a compact, rugged, and potentially portable instrument equipped with a control unit, sensors, actuators, and a pumping mechanism to process the liquid sample and reagents. The underlying, typically multi-branched channel architecture can usually not be properly washed to assure full regeneration of fluidic functionality, and also to avoid cross-contamination or carry-over of biosamples and reagents. Hence, in most cases, the chip is devised as single-use. The cost of material, equipment, process development, and machine time of this disposable, which is normally mass-produced by tool-based polymer replication schemes, such as injection molding, increases with the volume of bulk material and the surface area; in addition, the price tag on postprocessing, e.g., coatings, barrier materials, and reagents, as well as assembly steps, e.g., alignment of inserts and a lid, might be considerable, and may thus be commercially prohibitive for larger disc real estate.

Amongst various Lab-on-a-Chip technologies addressing comprehensive process integration of bioanalytical protocols, we investigate here liquid handling by centrifugal microfluidics that has been successfully advanced in industry and academia since the mid-1990s^14–28^ for various use cases, mostly in the context of biomedical in vitro diagnostics (IVD) for deployment at the PoC. Other applications comprise liquid handling automation for the life sciences, e.g., concentration/purification and amplification of DNA/RNA from a range of biosamples and matrices, process analytical techniques, and cell line development for biopharma, as well as monitoring the environment, infrastructure, industrial processes, and agrifood.

In most such “Lab-on-a-Disc” (LoaD) systems, biochemical assay kits are ported on the rotationally controlled scheme by dissecting the often conventional, possibly volume-reduced protocol into a sequence of Laboratory Unit Operation (LUOs) such as metering/aliquoting^29–31^, mixing^32–35^, incubation, purification/concentration/extraction^36,37^, homogenization^38,39^, particle filtering^40–45^, and droplet generation^46–48^. These LUOs are overwhelmingly processed in a batch-wise, rather than a continuous-flow fashion, by transiently sealing their fluidic outlet with a normally closed valve, thus intermittently stopping the flow while continuing rotation within certain boundaries, e.g., for vigorous agitation of the liquid sample. These centrifugal LUOs and their linked downstream detection techniques have been comprehensively reviewed elsewhere^24–26,49–65^.

The various, rotationally controlled centrifugal platforms analyzed in this work are predominantly distinguished by their valving mechanisms, which critically determine their capability for functional multiplexing^66^. Most of these “passive” flow control schemes root in the interplay of the centrifugal pressure exerted on a rotor-based liquid volume with a counteracting effect. Initial concepts were mainly based on interfacial tension to create burst valves or siphons primed by capillary action, which open by lifting^20^, lowering^15^ or accelerating^67^ the spin rate across critical frequency thresholds.

Yet, at least as stand-alone, such capillary valving mechanisms tend to be hard to fine control and to stabilize over the lifetime of the chip, ranging from production, packaging, storage, and transport to eventual handling by the user and processing on a PoC-compatible instrument; they also lack to provide a physical vapor barrier, hence making them unsuitable for longer-term onboard storage of liquid reagents as an important feature for many PoC scenarios.

To this end, several, normally closed valving schemes employing sacrificial barriers for retention of liquids and their vapor were introduced. In most of their implementations, the barrier is opened by an instrument- or rotor-based power unit^68^, e.g., for mechanical^69^, laser-^70–75^ or heat-induced perforation of a film^76^, melting of a wax plug^77–79^, or magnetic and pressure-induced deflection^80–82^, either during rotation or at rest. Also, passive, solvent-selective barriers have been explored^44,68,83^, which only transmit flow upon distinct physico-chemical stimuli.

More recently, centrifugo-pneumatic (CP) siphon valves were developed^30,67,84–86^ where the air is entrapped and centrifugally compressed by the incoming liquid during filling in a side chamber. Upon lowering the spin rate *ω*, the expansion of the pressurized volume pushes a surface-tension stabilized liquid “piston” within a microchannel across the crest point of their outlet siphon. This type of “Lab-on-a-Disc” (LoaD) platform uses a gas-impermeable, dissolvable film (DF), which is initially protected by a neighboring gas pocket. Once a geometry-dependent critical spin rate is surpassed, the forward meniscus wets the DF to, at the same time, vent the compression chamber and open a downstream outlet. Based on this conceptually simple CP-DF scheme, which can be solely controlled by the system-innate spindle motor, the integration of LoaD systems has been substantially elevated^83,87–89^.

This work will significantly support systematic layouts by providing a “digital twin”^90,91^, i.e., a virtual representation that serves as the real-time virtual, in silico counterpart of a physical object or process, for optimizing fluidic performance, robustness, packing density, and manufacturability of rotationally controlled valving schemes for LoaD platforms^92–95^. The first section surveys the fundamentals of centrifugal fields, continuity of mass and pressures contributing to hydrostatic equilibria at the core of valving liquid samples and reagents during batch-wise processing of their upstream LUOs. We then outline the concepts of critical spin rates and their associated bandwidths as quantitative, key performance indicators for systematically assessing the impact of experimental and geometrical tolerances on operational reliability at component- and system-level.

The next section covers the basic mechanisms underlying common, rotationally controlled valving technologies; we distinguish between high- and low-pass actuation, depending on whether they release their liquid upon increase or reduction of the spin rate, respectively. In addition to sacrificial barriers, capillary and pneumatic principles, various techniques for priming and thus opening siphon valves are surveyed. After pointing out their numerous synergistical benefits, we designate a full section on siphon valves that run against the pneumatic counter pressure into an outlet chamber that is initially sealed by a dissolvable-film (DF) membrane. Next, important performance metrics are defined, which guide the algorithmic design optimization^92,93^ towards fluidic LSI at high operational robustness before concluding with a comparison of passive valving techniques for LoaD platforms.

Note that, for convenience, the term “disc” will be used in general for designating the microfluidic, typically disposable device attached to the spindle motor. This alludes to the original idea to derive LoaD systems from common optical data storage technologies like CD or DVD. Yet, centrifugal microfluidic liquid handling does not depend on the outer shape of the usually polymeric “disc”, and many other formats, like mini-discs, segments, microscope slides, foils, or tubes, have been attached to the rotor in the meantime.

## Flow control

This paper focusses on rotationally controlled valving at the pivot of enhancing functional integration and reliability of centrifugal LoaD systems operating in “stop-and-go” batch mode between subsequent LUOs. We first look into the underlying general hydrostatic model before demonstrating its implementation for common centrifugal valving schemes.

### Centrifugal field

Under rotation at an angular frequency *ω* = 2*π* · *ν*, a particle of mass *m* experiences a centrifugal force $F_\omega = m \cdot {{{{{\mathcal{R}}}}}} \cdot \omega ^2$ with its center of mass located at the radial position ${{{{{\mathcal{R}}}}}}$. Within continuum mechanics underlying the modeling of fluidic systems, we consider the centrifugal force density

1 $$f_\omega = \varrho \cdot {{{{{\mathcal{R}}}}}} \cdot \omega ^2$$

which applies to a fluid distribution Λ of density $\varrho$. Note that in a suspension, $\varrho$ designates the difference of densities between the (bio-)particle and its surrounding medium.

Other pseudo forces (densities) arising in the non-inertial frame of reference, but of less relevance to this review, are the Euler force (density) $\left| {f_{{{{{\mathrm{E}}}}}}} \right| = \varrho \cdot {\mathcal{R}} \cdot {\mathrm{d}}\omega {{{{{\mathrm{/}}}}}}{\mathrm{d}}t$ pointing against the (vector of) the angular acceleration d*ω*/d*t*, and the Coriolis force (density) $|f_v| = 2\varrho \cdot \omega \cdot v$ acting on fluids moving at a (local) velocity *v*^49^; for common centrifugal systems, *f*_*v*_ aligns in the plane of the disc, and perpendicular to the flow, with its direction opposite to the sense of rotation^35,96,97^.

### Liquid distribution

More generally, we describe microfluidic systems by (contiguous) liquid segments of constant density $\varrho$, each containing a volume *U*_0,*i*_ which assume distributions {Λ_*i*_(*t*)} within a given structure Γ at a time-varying spin speed *ω*(*t*). In the (quasi) static approximations assumed in this work, i.e., very slow changes d*ω*/d*t* ≈ 0, we substitute the dependency on the time *t* by *ω*. Furthermore, for the sake of clarity, we look at each volume distribution Λ_*i*_(*ω*) individually, for which we apply the notation Λ(*ω*). In response to a centrifugal field *f*_*ω*_ (1), Λ(*ω*) assumes a radial extension Δ*r*(*t*) = *r* − *r*_0_ and mean radial position $\bar r\left( \omega \right) = 0.5 \cdot \left( {r + r_0} \right)$ between its confining upstream and downstream menisci *r*_0_ and *r*, respectively.

Expressed in cylindrical coordinates with the radial position *r* and the (potentially disjunct) local cross section *A*(*r*), the integral

2 $$U_0 = \mathop {\int}\limits_{{{\Lambda }}\left( \omega \right)} {{\mathrm{d}}V} = \mathop {\int}\limits_{\mathop{{\check{r}}}\limits\left( \omega \right)}^{\hat r\left( \omega \right)} {A\left( r \right)} {\mathrm{d}}r = {{{{{\mathrm{const}}}}}}.$$

corresponds to the total liquid volume *U*_0_ contained in the segment. The conservation of *U*_0_ requires that the volume between their inner- and outermost radial confinements $\mathop{{\check{r}}}\limits\left( \omega \right)$ and $\hat r\left( \omega \right)$, respectively, within the fixed structure Γ of cross-sectional function *A*(*r*) is preserved, i.e., d*U*_0_/d*ω* = 0. While Eq. (2) captures the general case of a randomly shaped liquid distribution Λ, we will later introduce simplified geometries, essentially composed of rectangular cuboids, for which the integral along the radial *r*-direction over Λ can be replaced by an analytical expression.

### Pressure contributions

#### Static pressures

Fluids shape according to the pressure distribution they are exposed to at a given location and time. The rotationally induced pressure head

3 $$p_\omega = {\it{\rho }} \cdot \bar r\Delta r \cdot \omega ^2$$

derives from (1), and scales with the mean radial position $\bar r = 0.5 \cdot \left( {r_0 + r} \right)$ and the radial extension Δ*r* = *r* − *r*_0_ of the liquid segment Λ(*ω*). The product

4 $$\bar r{{\Delta }}r = \frac{1}{2}\left[ {r + r_0} \right] \cdot \left[ {r - r_0} \right] = \frac{1}{2}\left[ {r^2 - r_0^2} \right]$$

in *p*_*ω*_ (3) can also be expressed by the front and rear radial positions of the menisci *r* and *r*_0_, respectively. For typical values $\varrho = 10^3\,{{{{{\mathrm{kg}}}}}}\,{{{{{\mathrm{m}}}}}}^{ - 3}$, $\bar r = 3\,{{{{{\mathrm{cm}}}}}}$, and Δ*r* = 1 cm, spin frequencies *ν* = *ω*/2*π* = 10 and 50 Hz roughly yield 12 and 300 hPa, respectively. So even for the faster rotational speeds *ω*, *p*_*ω*_ (3) only reaches about 1/3 of the standard atmospheric pressure *p*_std_ = 1013.25 hPa.

The pneumatic pressure

5 $$p_V = p_0 \cdot \frac{{V_0}}{V}$$

of a gas volume that is compressed from an initial volume *V*_0_ at *p*_0_ to *V* < *V*_0_ (law of Boyle–Mariotte) is of particular importance for this paper. By sufficient reduction of the final volume *V*, *p*_*V*_ (5) can, at least theoretically, assume randomly high values.

Also relevant to the small feature sizes in centrifugal microfluidics is the capillary pressure

6 $$p_{{\Theta }} = \frac{{4\sigma }}{D}\cos {{\Theta }}$$

as expressed for a liquid of surface tension *σ* in a channel (of round cross section) with a diameter *D* and the contact angle Θ between the liquid and the solid surface. For typical of values, e.g., *σ* = 72.8 mN m^−1^, Θ = 120° and a channel diameter *D* = 100 μm, the counterpressure *p*_Θ_ (6) can only sustain centrifugal pressure heads *p*_*ω*_ (3) in the range of *ν* = *ω*/2*π* ≈ 12 Hz.

#### Dynamic effects

In this work, we primarily look at the hydrostatic approximation d*ω*/d*t* ≈ 0 when dynamic pressure contributions are neglected. Yet, we briefly cover such effects on a semi-quantitative scale. During flow at a volumetric rate *Q* through a channel with round cross section *A* = *π* ⋅ (*D*/2)^2^, a pressure drop

7 $$p_{{{{{{Q}}}}}} = \frac{{64}}{\pi } \cdot \frac{{\eta \cdot L \cdot Q}}{D}$$

is experienced by a liquid of viscosity *η* across its axial extension *L* (law of Hagen–Poiseuille). For accelerating a liquid segment of volume *U* traveling at a speed *v* through a channel of cross section *A* at a rate d*v*/d*t* = *R* ⋅ d*ω*/d*t* with *Q* = *A* · *v*, a counterpressure

8 $$p_m = \frac{{\varrho \cdot U \cdot R \cdot {\mathrm{d}}\omega {{{{{\mathrm{/}}}}}}{\mathrm{d}}t}}{A}$$

is to be provided by a valve to stay closed. The rotationally induced local acceleration d*ω*/d*t* = *τ*_spindle_/*I*_disc_ is limited by the (maximum) torque *τ*_spindle_ of the motor, and the moment of inertia of the disc (and its rotor) *I*_disc_. For a solid disc of mass *m*_disc_, (homogenous) density $\varrho _{{{{{{\mathrm{disc}}}}}}} = {{{{{\mathrm{const}}}}}}.$ and radius *R*_disc_, we obtain $I_{{{{{{\mathrm{disc}}}}}}} \approx 0.5 \cdot m_{{{{{{\mathrm{disc}}}}}}} \cdot R_{{{{{{\mathrm{disc}}}}}}}^2$; however, strictly speaking, a LoaD cartridge exhibits cavities (with $\varrho _{{{{{{\mathrm{disc}}}}}}} \approx 0$) that are partially filled with a liquid distribution Λ = Λ(*t*) with a density $\varrho \ne \varrho _{{{{{{\mathrm{disc}}}}}}}$ and (a center of mass) moving radially outbound over time *t*.

#### Active flow control

Also externally powered and pneumatic controllers have been employed in centrifugal LoaD platforms^66,75,76,80,98–101^. The additional pressure *p*_ext_(*t*) has, for instance, been generated by external or rotor-based pressure sources and pumps^37,66,100^, by thermo-pneumatic actuation (law of Gay–Lussac), i.e., *p*_*T*_(*T*) ∝ *T*(*t*) (with the absolute temperature *T*)^31^, and by chemical reactions entailed by the expansion of gas volumes *V*(*t*), i.e., *p*(*t*) ∝ *V*(*t*)^101^. These techniques may readily be accounted for by including *p*_ext_(*t*) in the digital twin model. However, such active techniques tend to compromise the conceptual simplicity of the LoaD platform; rotationally controlled valving is thus the main focus of this paper.

### Hydrostatic equilibrium

For the batch-mode processing considered in the majority of centrifugal LoaD systems, flow is intermittently stopped by normally closed valves, i.e., the term *p*_*Q*_ ∝ *Q* (7) can be neglected. The spatial distribution of the liquid Λ(*ω*) is determined by the hydrostatic pressure equilibrium

9 $$\underbrace {\varrho \cdot \bar r{{\Delta }}r \cdot \omega ^2}_{p_\omega } + p_ \to = p_ \leftarrow$$

between *p*_*ω*_ ∝ *ω*^2^ (3), and further contributions *p*_→_ and *p*_←_ driving the liquid segment along or against the axial direction of the channel, respectively.

To trigger valving, the equilibrium distribution Λ resulting from (9) is modulated through at least one flexibly controllable pressure constituent *p*_*ω*_, *p*_→_, or *p*_←_. If the pressures *p*_→_ and *p*_←_ in the hydrostatic equilibrium (9) do not (explicitly) depend on *ω*, a spin rate

10 $$\omega = \sqrt {\frac{{p_ \leftarrow - p_ \to }}{{\varrho \cdot \bar r{{\Delta }}r}}}$$

can be attributed to a given Λ(*ω*) of a coherent liquid volume *U*_0_ within a structure Γ as a function of the radial product $\bar r{{\Delta }}r$ (4). A critical frequency *ω =* Ω is defined for Λ(Ω) representing the *ω*−boundary for retention of liquid, which is linked to a position of the front meniscus *r* = *r*(Ω). Note that for spin protocols *ω*(*t*) displaying steep ramps d*ω*/d*t* ≠ 0, the inertial term *p*_*m*_ (8) will have to be incorporated in *p*_←_ or *p*_→_ or, depending on whether the disc is accelerated (d*ω*/d*t* > 0) or slowed down (d*ω*/d*t* < 0), respectively.

#### Laboratory unit operations

In batch-mode-processing, valves need to close the outlet of an upstream LUO between the points in time of loading *T*_load_ and release *T*_open_, while agitating sample or reagents by a frequency protocol *ω*_LUO_(*T*_load_ < *t* < *T*_open_). Most LUOs, such as plasma separation from whole blood, run fastest and most efficiently at high centrifugal field strengths $f_\omega \propto {\mathcal{R}} \cdot \omega ^2$ (1) which, for a given layout Γ and its radial location ℛ, are established at high rates spin rates *ω*. Liquid retention is thus delineated by a threshold frequency Ω, and a resulting boundary for the field strength *f*_*ω*_(*ω* = Ω) from (1), for which the conditions ${{{{{\mathrm{max}}}}}}\left[ {\omega _{{{{{{\mathrm{LUO}}}}}}}\left( {t} \right)} \right] \,<\, {\hat \Omega}$ or ${\mathrm{min}}\left[ {\omega _{\mathrm{LUO}}\left( t \right)} \right] \,>\, {{{\check{\Omega}}}}$ need to be met for high-pass and low-pass valving, respectively.

Likewise, resilience of the valve to angular acceleration ramps ${{{{{\mathcal{R}}}}}} \cdot {\mathrm{d}}\omega {{{{{\mathrm{/}}}}}}{\mathrm{d}}t$ is important to agitate chaotic advection, as it is, for instance, required for liquid–liquid mixing^32^, incubation of dissolved biomolecules with surface-immobilized capture probes, resuspension of dry-stored reagents, or to support mechanical cell lysis through fixed-geometry obstacles or suspended (possibly magnetic) beads^102,103^.

Resulting, inertially induced pressure heads related to *p*_*m*_ (8) need to be factored into the calculation of the retention rates Ω. Also note that for supplying a given moment of inertia *I*_rotor_ of the rotor, such rotational acceleration |d*ω*/d*t*| ≠ 0 requires sufficient torque *τ*_spindle_ delivered by the spindle motor.

#### Actuation

For common rotational actuation by the spin rate *ω* through *p*_*ω*_ ∝ *ω*^2^ (3), the liquid segment is retained upstream of the valve until a certain frequency threshold $\omega = {{\Omega }} \in \left\{ {{{\hat{\mathrm \Omega }}}},{{{\check{\Omega}}}}\right\}$ is crossed, either surpassed ($\omega \, > \, {{{\hat{\mathrm \Omega }}}}$) or undershot ($\omega \, < \, {{{\check{\Omega}}}}$) for high-pass and low-pass valves, respectively. In some valving schemes presented later, the rotational actuation may not be achieved immediately after crossing Ω; proper (high-pass) valving is only assured once a (slightly) elevated actuation frequency Ω* > Ω is reached.

Alternatively, other, non-centrifugal pressure contributions to the equilibrium (9) may be modulated to prompt valving. Of particular interest for this work will be the venting of the compression chamber to level the pneumatic *p*_*V*_ (5) and atmospheric pressures *p*_0_, i.e., *p*_*V*_ ↦ *p*_0_, and normally *p*_0_ ≈ *p*_std_. Note also that in absence inbound pressure gradients, e.g., created by capillary pressure *p*_Θ_ (6) or active sources *p*(*t*), the center of gravity $\bar r$ (4) of the liquid distribution Λ may only move radially outwards due to the unidirectional nature of the centrifugal field *f*_*ω*_ (1) in the aftermath of valving.

### Reliability

#### Tolerances and bandwidth

Due to statistical deviations {Δ*γ*_*k*_} in its input parameters {*γ*_*k*_}, the experimentally observed retention frequency Ω (and Ω*) extends over an interval of standard deviation ΔΩ({*γ*_*k*_, Δ*γ*_*k*_}). In the digital twin concept presented here, the spread of the critical spin rate Ω (10)

11 $${{{{\Delta \Omega }}}}\left( {\left\{ {\gamma _k,{{\Delta }}\gamma _k} \right\}} \right) \approx \sqrt {\mathop {\sum}\limits_k {\left( {\frac{{\partial {{\Omega }}}}{{\partial \gamma _k}}{{\Delta }}\gamma _k} \right)} ^2}$$

can be calculated (and then systematically be optimized) by Gaussian error propagation, or through Monte-Carlo methods mimicking a large number of (virtual) test runs.

Using (11), we can directly relate the standard deviations ΔΩ in the critical spin rates Ω (10) to (the partial derivatives of) the fundamental experimental parameters {*γ*_*k*_} and their precision for the pipetting or metering *U*_0_, or for radial, vertical and lateral dimensions *R*, *d*, and *w*, resulting cross sections ${{\Delta }}A = \sqrt {\left( {w \cdot {{\Delta }}d} \right)^2 + \left( {d \cdot {{\Delta }}w} \right)^2}$ and (dead) volumes ${{\Delta }}V = \sqrt {\left( {wh \cdot {{\Delta }}d} \right)^2 + \left( {dh \cdot {{\Delta }}w} \right)^2 + \left( {dw \cdot {{\Delta }}h} \right)^2}$, delineating the valve structure Γ.

To avoid premature opening at $\omega \, < \, {{{\hat{\mathrm \Omega }}}}$ (or $\omega \, > \, \mathop{\Omega }\limits^{\smile}$ or in low-pass valving), the spin rate *ω* should be spaced by *M* · ΔΩ on either side of the nominal threshold value Ω, where *M* relates to the desired level of confidence; the aggregate rate of operational robustness *P*_*M*_ is mathematically evaluated by ${{{{{\mathrm{erf}}}}}}\big[M{{{{{\mathrm{/}}}}}}\sqrt 2 \big]$, with “erf” representing the error function; so, for *M* ∈ {1, 2, 3, 4, …}, valving reliability can be gauged at *P*_*M*_ ≈ {68%, 95%, 99.7%, 99.99%, …}. Hence, in the spirit of Six Sigma, these probabilities imply that, above *M* ≈ 6, the reliability of this (single) valving step is situated in the range of 1 to 10 defects per million opportunities (DPMO), for *M* ≥ 7, DPMOs are practically absent. The system-level reliability for *N* (independently operating) valves is calculated by (*P*_*M*_)^*N*^, e.g., $P_M^N \approx 77\%$ for *M* = 2 and *N* = 5.

#### Limited frequency space for multiplexing

The maximum degree of multiplexing is confined by the practically allowed range of spin rates *ω*^93^. At its lower end, the rotationally induced pressure head *p*_*ω*_ (3) still has to dominate capillary effects to keep the liquids at bay, which tends to require *ω* ≥ *ω*_min_ ≈ 2*π* ⋅ 10 Hz. On its upper end, motor power and concerns of lab safety may impose *ω* ≤ *ω*_max_ ≈ 2*π* ⋅ 60 Hz. Independent rotational actuation of concurrently loaded valves {*i*} requires non-overlapping bands {Ω_*i*_ ± *M* · ΔΩ_*i*_} (assuming Ω* ≈ Ω); the finite extent of the practical range *ω*_max_ − *ω*_min_ thus restricts the highest number of rotationally triggered sequential valving steps to *N* as calculated from $\omega _{{{{{{\mathrm{max}}}}}}} - \omega _{{{{{{\mathrm{min}}}}}}} \ge 2 \cdot M \cdot \mathop {\sum}\nolimits_{i = 1}^N {{{{{\Delta \Omega }}}}_i}$. Consequently, the available frequency envelope *ω*_min_ < *ω* < *ω*_max_ for fluidic multiplexing is best exploited by minimizing ΔΩ_*i*_, and to stagger the bands {Ω_*i*_ ± *M* · ΔΩ_*i*_} as closely as possible while avoiding overlap.

So, for example, a practically allowable *ω*-range within *ω*_min_ = 2*π* ⋅ 10 Hz ≤ *ω* ≤ *ω*_max_ = 2*π* ⋅ 60 Hz and a mean ΔΩ_*i*_/2*π* = 1 Hz, and a 99.99% reliability expressed by *M* = 4 at component level would imply an (average) bandwidth of 2 ⋅ *M* ⋅ ΔΩ_*i*_/2*π* = 2 ⋅ 4 ⋅ 1 Hz = 8 Hz, and thus provide proper operation of 50 Hz/8 Hz ≈ 6 concurrently loaded and serially triggered valving steps *i*; the reliability at system level would amount to 0.9999^3^ ≈ 99.97%. For *M* = 2, the width of the required frequency bands halves to provide space for of 50 Hz/4 Hz ≈ 12 frequency bands, at the expense of a drop in system-level robustness to 0.95^3^ ≈ 86%. Note that for the sake of simplicity, these back-of-the-envelope calculations were based on fixed ΔΩ_*i*_({*γ*_*k*_, Δ*γ*_*k*_}), while these standard deviations actually tend to broaden towards higher spin rates *ω*.

### Multiplexing

The digital twin approach will support the design of LoaD structures implementing multiplexed liquid handling protocols. Key flow control capabilities are the simultaneous and sequential release of several liquid volumes {*U*_*i,j*_} loaded to rotational valving structures {Γ_*i,j*_} located at radial positions {*R*_*i,j*_}. During their concurrent retention, the common spin rate needs to follow *ω* < min {Ω_*i*_ − *M* · ΔΩ_*i*_} for high-pass and *ω* > max {Ω_*i*_ + *M* · ΔΩ_*i*_} for low-pass valves. The order of release by venting simply relates to the sequence of the removal of the seals.

**Fig. 1:**
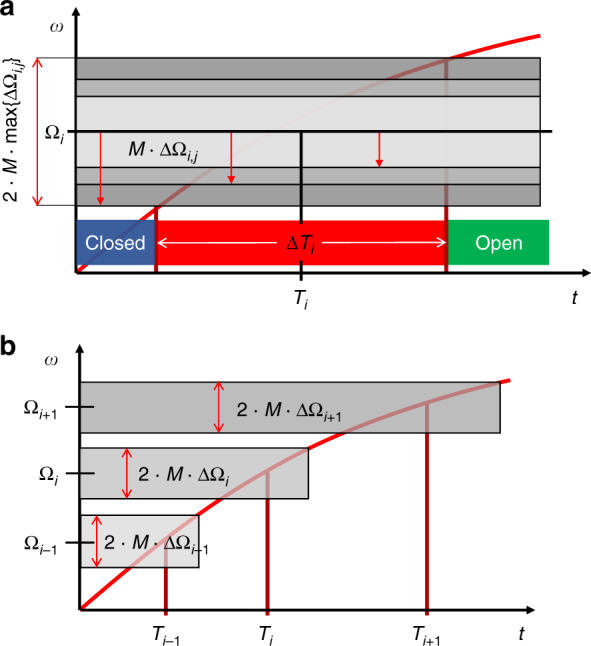
Fluidic multiplexing of rotationally actuated high-pass valving on the LoaD platform. **a** Robust concurrent actuation of valves {*i*, *j*} of a step *i* sharing the critical spin rate Ω_*i*_ at a time *T*_*i*_ requires lifting of the spin rate *ω* across the interval of width 2 ⋅ *M* ⋅ max{ΔΩ_*i*,*j*_}, which takes a time span Δ*T*_*i*_. **b** In serial actuation of steps {*i*}, the frequency intervals {Ω_*i*_ ± *M* ⋅ ΔΩ_*i*_} around the actuation frequencies {Ω_*i*_} need to be separated in order to assure the correct order of release at times {*T*_*i*_}. **a** Simultaneous actuation. **b** Sequential actuation.

For rotationally actuated, simultaneous release of high-pass valves {*i*, *j*} in the same step *i* at time *T*_*i*_ (Fig. 1a), the spin rate *ω*(*t*) needs to cross a zone min {Ω_*i,j*_ − *M* · ΔΩ_*i,j*_} < *ω* < max {${{\Omega }}_{i,j}^ \ast$ + *M* · Δ${{\Omega }}_{i,j}^ \ast$} centered at the (ideally identical) nominal critical rates Ω_*i*_ = {Ω_*i,j*_} and ${{\Omega }}_i^ \ast = \{ {{\Omega }}_{i,j}^ \ast \}$ within an interval Δ*T*_*i*_. For sequential actuation of valves {*i*} at times {*T*_*i*_} (Fig. 1b), the critical spin rates {Ω_*i*_} with Ω_*i*−1_ < Ω_*i*_ and *T*_*i*−1_ < *T*_*i*_, must be spaced so that (the outer boundaries of) their tolerance-related bands {Ω_*i*_ ± *M* ⋅ ΔΩ_*i*_} and $\{ {{\Omega }}_i^ \ast \pm M \cdot {{{{\Delta \Omega }}}}_i^ \ast \}$ do not overlap for all {*i*}.

## Basic centrifugal flow control schemes

### Sacrificial barriers

Apparently, straight-forward implementation of normally closed valves are removable materials for intermittently blocking liquids and gases. Various types of such sacrificial-barrier valves have been developed^104–106^. However, most of them require external actuation by an instrument-based module. Examples are wax plugs^71–73,78^ and barrier films that are disrupted by knife cutters (xurography)^69^, pressure sources, heat^71,77,107^, ice^108^, or (laser) irradiation^74^. Such flow barriers may be trivially included in the pressure equilibrium (9) by a counter pressure jumping to infinity when the liquid arrives at the sacrificial material.

In rotationally controlled, sometimes also referred to as “passive” LoaD systems that are mainly considered here, a sealing membrane opens once the rotationally induced pressure head $p_\omega \left( {R_{{{{{{\mathrm{seal}}}}}}}} \right) \propto {{\Omega }}_{{{{{{\mathrm{seal}}}}}}}^2 \, > \, p_{{{{{{\mathrm{seal}}}}}}}$ (3) applying at the location of the seal *R*_seal_ exceeds a minimum threshold *p*_seal_. Yet, the typically large magnitude and sensitivity of the release frequency Ω_seal_ on manufacturing tolerances {Δ*γ*_*k*_} tends to result in large spreads ΔΩ_seal_.

More recently, dissolvable films (DFs) that selectively disintegrate or become permeable upon contact with a specific solvent, e.g., of aqueous or organic nature, have been utilized for flow control^44,68,87^. It has been shown for a wider range of assays that the dissolved molecules do not interfere with bioanalytical protocols or detection, or, even if, could be effectively removed from the flow path into a side chamber under the prevalent laminar flow conditions. To provide timing of their upstream LUOs according to the programmable spin protocol *ω*(*t*), DF valves have been combined with centrifugo-pneumatic valving.

### Centrifugo-capillary burst valves

**Fig. 2:**
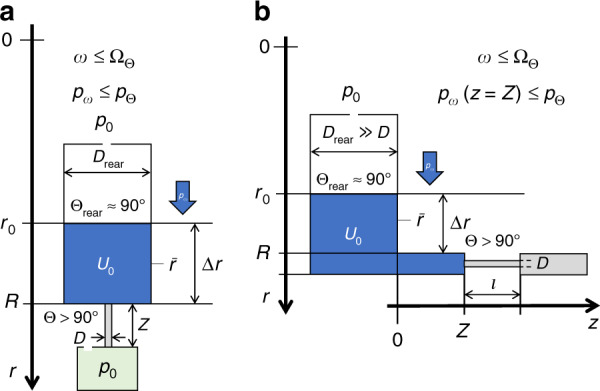
Centrifugo-capillary burst valve (not to scale). In this hydrophobic constriction, a centrifugal pressure head $p_\omega \propto \bar r{{\Delta }}r$ (3) runs against the capillary counterpressure *p*_←_ = *p*_Θ_ (6) calculated by the contact angles Θ > 90° and Θ_rear_ ≈ 90°, and their channel diameters *D* ≪ *D*_rear_ for their front and rear menisci, respectively. The valve stays closed until a critical spin rate Ω_Θ_ (12) is surpassed. (Left) Radial alignment. (Right) Isoradial alignment where radial “squeezing” of the liquid within the isoradial outlet may become an issue.

Hydrophobic constrictions, and also hydrophilic expansions with sharply defined edges, have been frequently used in centrifugal microfluidic system to stop the flow at a well-defined (axial) position *r* = *R* along a channel^20,49,50,54^. For a liquid segment driven by the centrifugal pressure *p*_*ω*_ (3) down a channel, such barriers exert a net counterpressure *p*_←_ composed of the capillary pressures *p*_Θ_ (6) of its radially outbound, front and rear menisci *p*_Θ,front_ and *p*_Θ,rear_, respectively (Fig. 2).

In the hydrostatic approximation (9), a threshold frequency for a hydrophobic constriction

12 $${{\Omega }}_{{\Theta }} = \sqrt {\frac{{4\sigma }}{{\varrho \cdot \bar r \cdot {{\Delta }}r}}\left( {\frac{{\cos {{\Theta }}_{{{{{{\mathrm{rear}}}}}}}}}{{D_{{{{{{\mathrm{rear}}}}}}}}} - \frac{{\cos {{\Theta }}}}{D}} \right)} \quad \approx \quad \sqrt { - \frac{{4\sigma }}{{\varrho \cdot \bar r \cdot {{\Delta }}r}}\frac{{\cos {{\Theta }}}}{D}}$$

is obtained from inserting *p*_←_ = *p*_Θ_(*D*, Θ) and *p*_→_ = *p*_Θ_(*D*_rear_, Θ_rear_) ≈ 0 (6) into (10), which needs to be exceeded for the liquid volume to progress to *r* > *R*. Note that with Θ > 90° for a hydrophobic coating, cos Θ < 0. Often, hydrophobic barriers are designed with *D*/*D*_rear_ ≪ 1 and/or Θ_rear_ ≈ 90°, so that the contribution from the rear meniscus becomes negligible. Assuming a density $\varrho = 1000\,{{{{{\mathrm{kg}}}}}}\,{{{{{\mathrm{m}}}}}}^{ - 3}$ and surface tension *σ* = 75 mN m^−1^ of water, its contact angle Θ ≈ 120° with Teflon, a mean radial position $\bar r = 3\,{{{{{\mathrm{cm}}}}}}$ and radial extension Δ*r* = 1 cm, and a constriction diameter *D* = 100 μm, we obtain threshold spin rates in the region of Ω_Θ_/2*π* ≈ 10 Hz. Note that at such low spin speeds *ω* < Ω_Θ_, detachment of a droplet, as outlined later in the context of the centrifugo-pneumatic valve in (15), is not expected as typically Ω_Θ_ ≪ Ω_drop_ (Fig. 2).

Hydrophilic expansions with Θ < 90° also produce a capillary stop. However, their retention frequencies Ω tend to be much smaller, and they sensitively depend on the exact shape, surface tension *σ* and contact angle Θ at the solid–liquid–gas interface. Similar geometrical features are thus often used for transient pinning of the meniscus, or, as so-called “phase guides” for shaping the front of creeping flows, e.g., during capillary priming of microfluidic chips.

Moreover, note that both types of capillary valves do not to curb evaporation, which leads to volume loss and exposure of the connected fluidic network to humidity; these valves are thus unsuitable for use in longer-term liquid storage. Also, capillary barriers often involve significant manufacturing and assembly challenges, as all four walls, with one of them usually represented by a flat lid, need to display homogeneous, well-localized coatings. Otherwise, retention rates Ω might shift, or flow might still creep, instead of being cleanly halted, as required for proper batch-mode processing.

### Centrifugo-pneumatic burst valves

#### Pneumatic retention

**Fig. 3:**
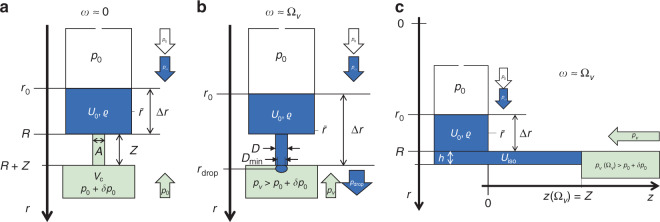
Centrifugo-pneumatic valving (not to scale). **a** At rest (*ω* ≈ 0), the liquid stops at *r* = *R* in front of the radial outlet of length *Z* and a sufficiently narrow cross section *A*, which is followed by a compression chamber. At this point, the gas volume *V*_C_ + *A* · *Z* is at ambient pressure *p*_0_, plus a small contribution *δp*_0_ with *χ* = *δp*_0_/*p*_0_ ≪ 1 linked to (dynamic) filling effects. **b** At a critical spin rate Ω_*V*_, the meniscus protrudes to *r* = *R* + *Z* at the transition to the pneumatic chamber. Now the pneumatic counterpressure has increased to *p*_*V*_ = *p*_0_⋅(1 + *χ*) ⋅ (*V*_C_ + *A* ⋅ *Z*)/*V*_C_, and a droplet of volume *V*_drop_ ≈ (4/3)*π*(*D*/2)^3^ located at *r* = *r*_drop_ ≈ *R* + *Z* emerges from the orifice. Centrifugo-pneumatic valving is triggered once the weight force $F_m = V_{{{{{{\mathrm{drop}}}}}}} \cdot f_\omega$ (1) of the droplet exceeds the counteracting surface tension force *F*_*σ*_ = *πD*_min_⋅*σ* at its minimum diameter *D*_min_ = *D*/*κ* with *κ* > 1. While the exact dynamics of its detachment are unclear and hard to quantify, it is assumed that the hydrodynamic agitation caused by the detaching droplet disrupts the integrity of the liquid plug, thus causing successive release of the entire liquid into the compression chamber, while gas gradually escapes in the reverse direction to atmosphere. **c** CP valving with an isoradial outlet pinned at *r* = *R*. A minimum liquid volume *U*_0_ ≥ *U*_iso_ is required for generating Δ*r* > 0, and thus *p*_*ω*_ > *p*_*V*_(Ω_*V*_) for opening. Towards high field strength *f*_*ω*_ (1), the shape of the front meniscus progressively distorts. **a** Filling at rest. **b** State at critical spin rate ${{\Omega }}_V$. **c** Isoradial configuration.

For rotational flow control, the centrifugal pumping by *p*_*ω*_ (3) can be opposed by a pneumatic pressure *p*_←_ = *p*_*V*_ (5) arising from the compression of a gas volume from *V*_0_ to *V* < *V*_0_ enclosed at the downstream end of the structure Γ. As outlined in Fig. 3a, this counter pressure *p*_*V*_ may differ from its initial value *p*_0_ + *δp*_0_ = *p*_0_ ⋅ (1 + *χ*) at *ω* ≈ 0; the small offset *δp*_0_ with *δp*_0_/*p*_0_ = *χ* ≪ 1 of the gas pressure *p*_0_ at the volume *V*_C_ + *A* · *Z* represents a departure from the hydrostatic approximation attributed to dynamic effects during filling. It may be explained by air that is drawn with the flow of liquid into the compression chamber, and either needs to be quantified empirically, or by advanced simulation.

At *ω =* Ω_*V*_ (Fig. 3b), the original gas volume is reduced by *A* · Z to *V*_C_, thus increasing its pressure to *p*_*V*_ = *p*_0_⋅(1 + *χ*)⋅(*V*_C_ + *A*⋅*Z*)/*V*_C_ (5). The cross section *A* needs to be sufficiently small so that the surface tension sustains “piston-like” characteristics of the liquid plug. Under these conditions, we set

13 $$p_ \leftarrow = p_V = p_0 \cdot \left( {1 + \chi } \right) \cdot \left( {1 + \frac{{A \cdot Z}}{{V_{{{{{\mathrm{C}}}}}}}}} \right)$$

and *p*_→_ = *p*_0_ to obtain a critical spin rate (10)

14 $${{\Omega }}_V = \sqrt {\frac{{p_0}}{{\varrho \cdot \bar r \cdot {{\Delta }}r}} \cdot \left[ {\left( {1 + \chi } \right) \cdot \left( {1 + \frac{{A \cdot Z}}{{V_{{{{{\mathrm{C}}}}}}}}} \right) - 1} \right]} \approx \sqrt {\frac{{p_0}}{{\varrho \cdot \bar r \cdot {{\Delta }}r}} \cdot \frac{{A \cdot Z}}{{V_{{{{{\mathrm{C}}}}}}}}}$$

to position the front meniscus at *r* = *R* + *Z*. For typical values, $\bar r \approx R = 3\,{{{{{\mathrm{cm}}}}}}$, Δ*r* = 1 cm, a volume ratio *A* · Z/*V*_C_ ≈ 1/10 and *δp*_0_ ≈ 0, this estimate provides a release threshold in the region of Ω_V_/2*π* ≈ 22 Hz. An isoradial variant of the valve (Fig. 3c) tends to display a tilted meniscus surface, thus compromising the validity of the formula for Ω_*V*_ (14) towards large *ω*.

#### Droplet release

To effectuate basic centrifugo-pneumatic valving, a droplet of volume *V*_drop_≈(4/3)*π*(*D*/2)^3^ ≪ *V*_C_ located at the radial position *r*_drop_ ≈ *R* + *Z* is pulled by the centrifugal force $F_m = V_{{{{{{\mathrm{drop}}}}}}} \cdot f_\omega = V_{{{{{{\mathrm{drop}}}}}}} \cdot \varrho \cdot r_{{{{{{\mathrm{drop}}}}}}} \cdot \omega ^2$ (1) out of the orifice to the compression chamber. While the exact mechanism is somewhat obscure, we consider a simplified model akin to goniometric measurement of surface tension; detachment of the hanging droplet is suppressed until its surface tension force *F*_*σ*_ = *σ* ⋅ *πD*_min_ applying at its minimum cross section of diameter *D*_min_ = *D*/*ε* with *ε* > 1 cannot support its weight force $F_m \approx \varrho \cdot V_{{{{{{\mathrm{drop}}}}}}} \cdot r_{{{{{{\mathrm{drop}}}}}}} \cdot \nu ^2$ anymore. This model leads to a critical spin rate

15 $${{\Omega }}_{{{{{{\mathrm{drop}}}}}}} \approx \sqrt {\frac{{\sigma \cdot \pi D_{{{{{{\mathrm{min}}}}}}}}}{{\varrho \cdot \left( {4/3} \right)\pi \left( {D/2} \right)^3 \cdot r_{{{{{{\mathrm{drop}}}}}}}}}} \approx \frac{1}{D}\sqrt {\frac{{6\sigma }}{{\varepsilon \cdot \varrho \cdot \left( {R + Z} \right)}}}$$

for droplet release with *D*_drop_ ≈ *D*.

Inserting typical values *D* ≈ 200 μm, *σ* ≈ 75 mN m^−1^, *ε* ≈ 1.5, $\varrho \approx 1000\,{{{{{\mathrm{kg}}}}}}\,{{{{{\mathrm{m}}}}}}^{ - 3}$ and *R* ≈ 3 cm in (15), we obtain Ω_drop_/2*π* ≈ 80 Hz. This very coarse “back of the envelope” calculation reveals that the threshold spin rate for droplet release Ω_drop_ (15) sensitively depends on the diameter of the outlet *D*.

#### Compensation of ambient pressure

The main systematic error in the threshold spin rate Ω_*V*_ (14) is introduced by its dependence on the actual ambient (atmospheric) pressure *p*_0_ from its nominal (standard) value *p*_std_ = 1013.25 hPa at sea level, which remains rather constant at a given geolocation, and over the course of a bioassay, typically minutes to an hour. By timely local measurement of *p*_0_, e.g., by a commodity pressure sensor mounted to the instrument, the spin protocol *ω*(*t*) can be flexibly adjusted by the factor $\sqrt {{{\Delta }}p_0{{{{{\mathrm{/}}}}}}p_{{{{{{\mathrm{std}}}}}}}}$ to compensate the dependency Ω = Ω(*p*_0_).

**Fig. 4:**
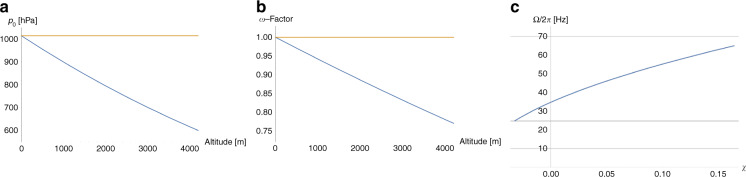
Variation in the atmospheric pressure *p*_0_. **a** Barometric formula quantifies the decrease in atmospheric pressure from sea level to roughly the limit of habitable space on earth at roughly 4000 m altitude. **b** Compensation factor of the spin rate *ω* for altitude adjusted (standard) pressure *p*_0_. For the given example, the critical spin rate would need to be lowered by about 20% from Ω/2*π* = 25 Hz at sea level to about 20 Hz in high altitude. In general, any known local pressure *p*_0_, either caused by altitude or weather, can be flexibly accommodated by adjusting the spin rate *ω* according to $\sqrt {p_0{{{{{\mathrm{/}}}}}}p_{{{{{{\mathrm{std}}}}}}}}$ (23). **c** Shift of the retention rate Ω (23) with the (dimensionless) coefficient *χ* (14) representing potential dynamic effects entailing deviations of the effective pressure in the gas volume enclosed in the compression chamber from ambient *p*_0_ at the point when it is pneumatically isolated from the liquid in the isoradial channel. **a** Changes of atmospheric pressure *p*_0_ with altitude with respect to *p*_std_. **b** Adjustment factor for the retention rate ${{\Omega }}$ (23) to compensate altitude. **c** Impact of dynamic filling effects characterized by the coefficient $\chi$ on the retention rate ${{\Omega }}$ (23).

Figure 4a shows the reduction of the atmospheric pressure with altitude up to the highest human settlements by about 30% (left), and the required compensation of the spin rate *ω* to assure proper retention of liquid volumes by about 3%, 6%, 9%, and 12% at 500 m, 1000 m, 1500 m, and 2000 m, respectively (Fig. 4b). Note that a tolerance-forgiving design would then make sure that the (lower) centrifugal field *f*_*ω*_ (1) would still be sufficient to carry out the upstream LUO, possibly by also extending the length of its correlated time interval *T*_open_ − *T*_load_ in the spin protocol *ω*(*t*).

Similar considerations can be applied for the compensation of *χ* ≠ 0 (14). As portrayed in Fig. 4c, the valve geometry should either be tuned to widely suppress such dynamic effects, i.e., *χ* ≈ 0, or to at least stabilize *χ*, i.e., Δ*χ* ≈ 0; a finite, but constant *χ* can thus be accounted for by an adjusted spin rate protocol *ω*(*t*), as already described above for compensating deviations of the local ambient from standard atmospheric pressure *p*_std_.

#### Rotational actuation

Followingly, droplet release triggering the opening of centrifugo-pneumatic valves essentially proceeds at frequencies

16 $${{\Omega }}_{{{{{{\mathrm{cpv}}}}}}} = {{{{{\mathrm{min}}}}}}\left\{ {{{\Omega }}_V,{{\Omega }}_{{{{{{\mathrm{drop}}}}}}}} \right\}$$

and may be associated with rather large uncertainties ΔΩ_cpv_ caused by effects that are hard to quantify by the simple (hydrostatic) modeling presented here.

It is surmised that the detachment of a (first) hanging drop above Ω_cpv_ (16) severely disrupts the surface of the liquid plug, so that a certain portion of the compressed air can escape through the narrow outlet, and thus gradually vent the compression chamber. This partial pressure release has a bigger impact on the pneumatic counter pressure *p*_*V*_ (5) than the loss of liquid volume to the chamber on the radial product $\bar r{{\Delta }}r$ in *p*_*ω*_ (3). Consequently, more liquid will protrude into the compression chamber to progressively complete the transfer. Such step-wise liquid transfer has indeed been experimentally observed (qualitatively) in the region *ω* ≈ Ω_cpv_. It was accompanied by a large spread ΔΩ_cpv_, which may reflect the sensitivity of Ω_drop_ (15) to is experimental input parameters.

Their comparatively high burst frequencies Ω_cpv_ in (16), combined with their large spread ΔΩ_cpv_, make such basic centrifugo-pneumatic flow control schemes mainly suitable for final valving steps into a dead-ended cavity, e.g., for aliquoting of liquid sample or reagents into detection chambers^109^. Moreover, centrifugo-pneumatic valving requires powerful spindle motors, aerodynamic optimization, and mechanically well-balanced rotors, and may raise concerns about lab safety.

#### Venting

**Fig. 5:**
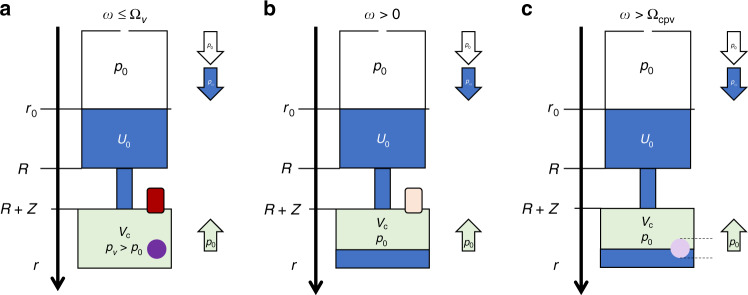
Centrifugo-pneumatic valving by venting. **a** Below the retention rate *ω* ≤ Ω_*V*_ (14), the liquid is kept outside the pneumatic chamber, which is closed by gas-impermeable or dissolvable film (DF) membranes. **b** Upon its dissolution, the pneumatic counter pressure *p*_*V*_ converges to the atmospheric pressure *p*_0_, thus releasing the liquid at any *ω* > 0. **c** For this high-pass valve, liquid enters the compression chamber above a retention frequency Ω_cpv_ (16). After a sufficient volume *U*_DF_ = *β* · *V*_C_ with 0 < *β* < 1 has entered the compression chamber of (dead) volume *V*_C_, the liquid wets and thus opens the DF to trigger flow at any *ω* > 0 through an outlet, which is, e.g., located in a lower layer connected through a vertical via concealed underneath the DF. **a** Retention phase. **b** Venting through membrane. **c** Venting through dissolvable film.

Opening the compression chamber to atmosphere, i.e., *V*_C_ ↦ ∞ leading to *p*_*V*_ ↦ *p*_0_ (5), constitutes an alternative actuation mechanism for these CP-valves. While this principle would allow high retention frequencies Ω_*V*_ (14), and thus vigorous agitation for its upstream LUO (Fig. 5a), it turned out to be challenging to provide a conceptually simple mechanism for perforating the pneumatic chamber during high-speed rotation (Fig. 5b). Especially in the context of “event-triggered” valving concepts^88,89,110^, venting of compression chambers, which are initially by sealed dissolvable film (DF) membranes, has been implemented through arrival of a sufficient volume of ancillary liquid at strategic locations on the disc (Fig. 5c).

### Centrifugal siphon valving

#### Layout and liquid distribution

**Fig. 6:**
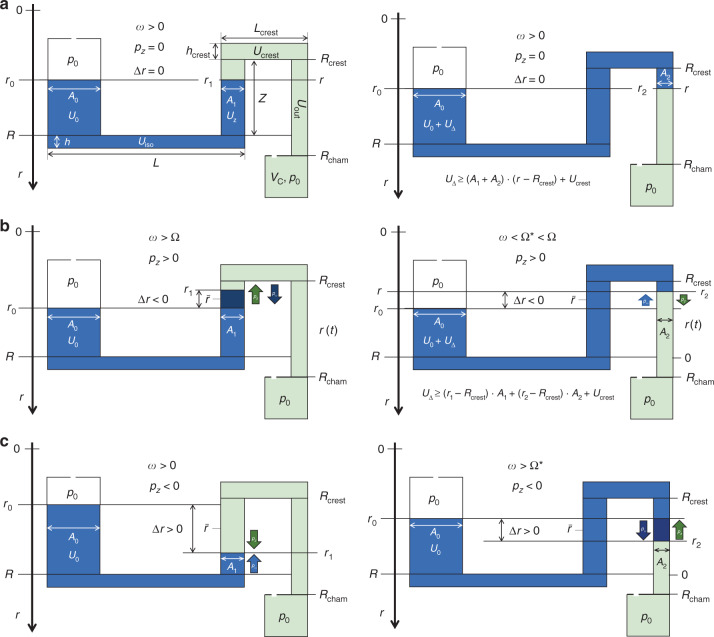
Centrifugally controlled siphon valving with distributions Λ for retention (left) and release (right) (linearized display, not to scale). The layout Γ features an inlet reservoir of cross section *A*_0_ and a bottom at *R*, connected to an isoradial outlet of length *L*_iso_, radial height *h*_iso_ and volume *U*_iso_. The following siphon channel starts with an inbound segment of radial length *Z* and cross section *A*_1_ between *R* and a crest point *R*_crest_ = *R* − *Z*, an inner isoradial channel of axial length *L*_crest_, radial height *h*_crest_ and volume *U*_crest_, and an outlet channel of cross section *A*_2_ and volume *U*_out_ along the interval *R*_crest_ < *r* < *R*_cham_ leading to a collection chamber at *R*_cham_ > *R*. The ambient pressure *p*_0_ in the vicinity of *p*_std_ applies to all vented chambers. The meniscus positions confining the liquid *U*_0_ in the inlet, the inbound and outbound sections are *r*_0_(*ω*), *r*_1_ = *r*(*ω* = Ω) and *r*_2_ = *r*(Ω^*^), respectively. A net centrifugal pressure *p*_*ω*_ ∝ Δ*r*⋅*ω*^2^ (3) with Δ*r*(*ω*) = *r*_*i*_(*ω*) − *r*_0_(*ω*) and *i* ∈ {1, 2} plus an axially directed pressure difference *p*_*z*_ = *p*_→_ − *p*_←_ (assumed here as constant along the axial direction), which is composed of forward and backwards contributions *p*_→_ and *p*_←_, shape the liquid distribution Λ(*ω*). A first critical retention frequency Ω is set with *p*_*ω*_(Ω) + *p*_*z*_(Ω) = 0 at *R*_crest_ < *r*_1_ < *R* in the inbound segment. Ω is usually chosen so that *r*_1_(Ω) settles sufficiently below *R*_crest_ to account for *M* · ΔΩ (11) linked to tolerances {Δ*γ*_*i*_}. A second critical position *r*_2_ is found in the outlet segment at a second spin rate Ω* at *p*_*ω*_(Ω*) + *p*_*z*_(Ω*) = 0, possibly after adding a liquid volume *U*_Δ_ to *U*_0_ (**a**). Valving is practically possible if the calculated Ω and Ω* reside within the frequency envelope between *ω*_min_ and *ω*_max_, suitable *U*_0_ > *U*_iso_ and Γ, so the radial positions *r*_0_, *r*_1_ and *r*_2_ are located within their allowed radial intervals *R*_min_ < *r*_0_ < *R*, *R*_crest_ < *r*_1_ < *R* and *R*_crest_ < *r*_2_ < *R*_cham_. **a** Priming by volume addition. For $p_ \to = p_ \leftarrow = p_0$, and thus $p_z = 0$, and ${{\Delta }}r = 0$ for all spin rates $\omega \, > \, 0$. The front meniscus resides at $r = r_1 \, > \, R_{{{{\mathrm{crest}}}}}$ in the inbound segment of cross section $A_1$ for all liquid volumes $U_0$ within $0 \, < \, U_0 - U_{{{{\mathrm{iso}}}}} \, < \, \left( {A_0 + A_1} \right) \cdot Z$. Then, an amount $U_{{\Delta }} \, > \, 0$ with $U_0 + U_{{\Delta }} = (A_0 + A_1) \cdot Z + U_{{{{\mathrm{iso}}}}} + U_{{{{\mathrm{crest}}}}} + A_2 \cdot (r_2 - R_{{{{\mathrm{crest}}}}})$ needs to be added to shift the meniscus across the crest channel of volume $U_{{{{\mathrm{crest}}}}}$ to $r_2 \, > \, R_{{{{\mathrm{crest}}}}}$ in the outbound channel of cross section $A_2$. The conservation of liquid volume $U_0$ between ${{\Omega }}$ and ${{\Omega }}^ \ast$ demands $U_0 = A_0 \cdot \left[ {R - r_0\left( {{\Omega }} \right)} \right] + U_{{{{\mathrm{iso}}}}} + A_1 \cdot \left[ {R - r_1\left( {{\Omega }} \right)} \right] = A_0 \cdot \left[ {R - r_0\left( {{{\Omega }}^ \ast } \right)} \right] + U_{{{{\mathrm{iso}}}}} + A_1 \cdot Z + U_{{{{\mathrm{crest}}}}} + A_2 \cdot \left[ {R_{{{{\mathrm{crest}}}}} - r_2\left( {{{\Omega }}^ \ast } \right)} \right] - U_{{\Delta }}$. After a critical position $R_{{{{\mathrm{crest}}}}} \, < \, r_2 = r_0 \, < \, R_{{{{\mathrm{cham}}}}}$ is reached, ${{\Delta }}r \, > \, 0$ holds for all $\omega \, > \, 0$ without further volume addition $U_{{\Delta }}$, so centrifugally driven forward pumping into the vented receiving chamber situated at $R_{{{{\mathrm{cham}}}}} \, > \, R$ kicks in. **b** Priming of low-pass siphon valves occurs for $p_z \, > \, 0$. (Left) At the retention rate $\omega = {{\Omega }}$, the front meniscus resides in the inbound section of cross section $A_1$ at $r_1$ with $R_{{{{\mathrm{crest}}}}} \, < \, r_1 \, < \, R$, so that ${{\Delta }}r \, < \, 0$ and $p_\omega = p_z$. (Right) At a second hydrostatic equilibrium defining release at ${{\Omega }}^ \ast \, < \, {{\Omega }}$ $\left( {{{{\mathrm{and}}}}\;U_{{\Delta }} = 0} \right)$, the meniscus has passed the crest channel of volume $U_{{{{\mathrm{crest}}}}}$ to arrive at a second position $r_2$ in the radial outlet of cross section $A_2$, so that for $\omega \, < \, {{\Omega }}^ \ast$, $p_\omega$ aligns parallel to the axial direction and $p_z$, hence constructively pumping the liquid into the (vented) outer recess at $R_{{{{\mathrm{cham}}}}} \, > \, R$. **c** Priming of high-pass siphon valves with $p_z \, < \, 0$. (Left) During retention $\left( {\omega \, < \, {{\Omega }}} \right)$ of the meniscus at $r_1$ in the inbound section of cross section $A_1$ with $R_{{{{\mathrm{crest}}}}} \, < \, r_0 \, < \, r_1 \, < \, R$, a liquid level difference ${{\Delta }}r \, > \, 0$ is needed to compensate the counterpressure $p_z$. (Right) For $\omega = {{\Omega }}^ \ast$ $\left( {{{{\mathrm{and}}}}\;U_{{\Delta }} = 0} \right)$, a critical point $R_{{{{\mathrm{cham}}}}} \, > \, r_2 \, > \, R_{{{{\mathrm{crest}}}}}$ is reached in the radially outbound channel of cross section $A_2$. For any $\omega \, > \, {{\Omega }} \ast$, the centrifugal pressure $p_\omega$ (3) exceeds $p_z$ to transfer the liquid into the (vented) outlet at $R_{{{{\mathrm{cham}}}}}$. Note that for pneumatically controlled principles, i.e., $p_ \leftarrow = p_V$, the receiving chamber needs to be sealed.

For understanding the core principle underlying centrifugal siphoning, Fig. 6 displays a basic design Γ with an inner reservoir of cross section *A*_0_, and a bottom at *R* which is connected by an isoradial segment of volume *U*_iso_ of radial length *L*_iso_ and height *h*_iso_ to a siphon channel of (constant) cross section *A* < *A*_0_. Its isoradial section at the crest point *R*_crest_ = *R* − *Z* has a volume capacity *U*_crest_, axial length *L*_crest_ and radial height *h*_crest_. The radially directed outlet channel of volume *U*_out_ extends between *R*_crest_ and the final receiving chamber starting at *R*_cham_ > *R*. All parts of the siphon structure up to the crest point are, for the sake of simplicity, chosen to have the same depth *d*, typically 1 mm, and a small fraction of that beyond that point.

We consider adjustment of the liquid distribution Λ(*ω*) between (hydrostatic) equilibria *p*_*ω*_(*ω*) + *p*_*z*_(*ω*) = 0, with *p*_*ω*_ from (3), and an axially directed pressure head *p*_*z*_ = *p*_→_ − *p*_←_, with forward and reverse contributions *p*_→_ and *p*_←_, respectively, in response to a (slowly) changing spin rate *ω = ω*(*t*). A first equilibrium distribution Λ(Ω) can be found in the inbound segment at *r* = *r*_1_ with *R*_crest_ < *r*_1_ < *R* for a loaded liquid volume *U*_0_ > *U*_iso_. The retention rate Ω is usually set so that the meniscus *r*_1_ stays well below *R*_crest_ to factor in a safety margin *M* · ΔΩ (11) resulting from tolerances {Δ*γ*_*i*_} in the input parameters {*γ*_*i*_}, and the targeted level of reliability denoted by *M*. Optionally, the meniscus in the inbound segment may be “pinned” to a fixed target position *r*_1_ by a low capillary barrier, or by a local widening of the channel cross section (which would only slightly change the following calculations).

A second critical point *R*_crest_ < *r*_2_ = *r*(Ω*) < *R*_cham_ is situated in the outbound channel beyond which any further increase in Δ*r* = *r*_2_(*ω*) − *r*_0_(*ω*), e.g., induced by topping up a liquid volume *U*_Δ_ or modulating *ω*, leads to a growth in Δ*r*, and hence the pumping force *p*_*ω*_ (3). Different types of siphon valves can be categorized by their priming mechanism to assure *p*_*ω*_(*ω*) + *p*_*z*_(*ω*) > 0 for migrating between *r*_1_ = *r*(Ω) in the inbound and *r*_2_ = *r*(Ω*) in the outbound segments.

#### Priming

In volume addition mode, priming is triggered by topping up *U*_0_ with *U*_Δ_ > 0. Figure 6a shows the simplest case for *p*_*z*_ = 0, so pumping initiates at any spin rate *ω >* 0 once the outlet channel is reached to assure Δ*r* > 0, so that the liquid level *r* in the radially outbound channel has fallen below the inner meniscus in the inlet reservoir *r*_0_, i.e., *r > r*_0_.

For low-pass siphon valving (Fig. 6b), *p*_*z*_ > 0 and *U*_Δ_ = 0, a threshold Ω* < Ω can be determined to guarantee pumping for *ω* < Ω*. Conversely, according to the basic high-pass siphoning concept illustrated in Fig. 6c, liquid is released when *p*_*ω*_ + *p*_*z*_ > 0 along the entire path of the front meniscus to the end of the outlet at *R*_cham_, and during release into the chamber.

Note that, for a given design Γ, the liquid distributions Λ(*ω*) need to obey the continuity of volume (2) as expressed by *A*_0_ ⋅ [*R* − *r*_0_(Ω)] + *U*_iso_ + *A*_1_ ⋅ [*R* − *r*_1_(Ω)] = *A*_0_ ⋅ [*R* − *r*_0_(Ω*)] + *U*_iso_ + *A*_1_ ⋅ *Z* + *U*_crest_ + *A*_2_ ⋅ [*r*_2_ − *R*_crest_] − *U*_Δ_ (Fig. 6). Moreover, for valid solutions Λ(Ω) and Λ(Ω*), the menisci at *r*_*i*_ with *i* ∈ {0, 1, 2} need be situated within the corridors *R*_min_ < *r*_0_ < *R*, *R*_crest_ < *r*_1_ < *R*, and *R*_crest_ < *r*_2_ < *R*_cham_, while *ω*_min_ ≤ *ω* ≤ *ω*_max_ needs to hold for both critical spin rates *ω =* Ω and *ω =* Ω*.

#### Pneumatic priming

**Fig. 7:**
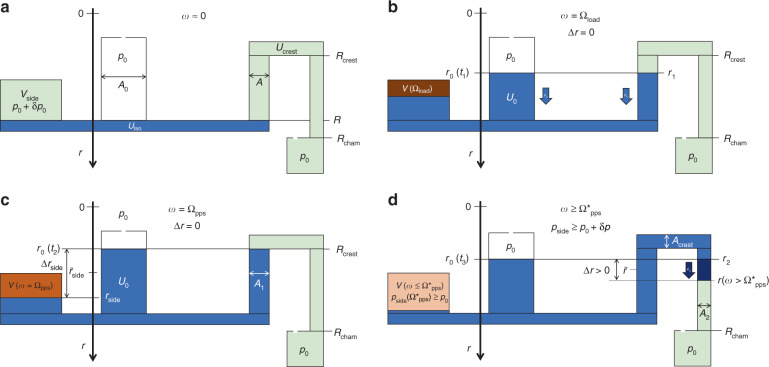
Pneumatic siphon priming with *p*_*z*_ = *p*_→_ = *p*_*V*_ and a vented outlet, i.e., *p*_←_ = *p*_0_ (not to scale). **a** Liquid is loaded at ${{t}_{0}}$ and $\omega \approx 0$ to the inlet so the air in the side chamber of dead volume $V_{{{{\mathrm{side}}}}}$ is cut off from atmosphere at $p_0$ at an initial pressure $p_0 + \delta p_0 = p_0 \cdot \left( {1 + \chi } \right)$ with $\chi \ll 1$. **b** At the highest spin rate $\omega = {{\Omega }}_{{{{\mathrm{load}}}}}$, the gas pocket is centrifugally compressed to ${V}\left( {{{\Omega }}_{{{{\mathrm{load}}}}}} \right) \,<\, {V}_{{{{\mathrm{side}}}}}$ while assuring ${r}_1(\omega ) = {r}_2(\omega ) \,>\, {R}_{{{{\mathrm{crest}}}}}$. **c** At $\omega = {{\Omega }}_{{{{\mathrm{pps}}}}} \, < \, {{\Omega }}_{{{{\mathrm{load}}}}}$, the local pressure to $p_{{{{\mathrm{side}}}}} = p_0 \cdot \left( {1 + \chi } \right) \cdot V_{{{{\mathrm{side}}}}}/V\left( {{{\Omega }}_{{{{\mathrm{pps}}}}}} \right)$ sets the common liquid levels ${r} = {r}_0 = {R}_{{{{\mathrm{crest}}}}}$ in the inlet and inbound segment of cross section ${{A}_{1}}$, i.e., ${{\Delta }}r = 0$. **d** Once $\omega \,<\, {{\Omega }}_{{{{\mathrm{pps}}}}}^ \ast \,<\, {{\Omega }}_{{{{\mathrm{pps}}}}}$ has been adequately lowered, the front meniscus has passed the crest channel at ${R}_{{{{\mathrm{crest}}}}}$ of cross section $A_{{{{\mathrm{crest}}}}}$ to reach the second equilibrium position at $r_2$ in the outbound channel of cross section $A_2$, i.e., ${{r}_{2} = {r}\left( {\omega = {{\Omega }}_{{{{\mathrm{pps}}}}}^ \ast } \right)}$ For sufficiently small ${{A}_{1}}$, $A_{{{{\mathrm{crest}}}}}$ and $A_2$, liquid is centrifugally “pulleyed” from the inlet through the siphon channel into the outlet chamber commencing at $R_{{{{\mathrm{cham}}}}}$.

The same principle used for generating the counterpressure *p*_←_ in the basic pneumatic valving mode (Fig. 3) can also be sourced for priming the siphon valve^84,111^, i.e., *p*_→_ = *p*_*V*_ (5). To this end, a side chamber of dead volume *V*_side_ is laterally connected to the inlet reservoir (Fig. 7). In a (somewhat idealized) multi-step procedure, a first liquid volume *U*_iso_ is loaded at small *ω* ≈ 0 (Fig. 7a). At this stage, a gas volume *V*_side_ of the same size as the side chamber is disconnected from the main valving structure by the incoming liquid, which experiences a pressure *p*_0_ + *δp*_0_, with *δp*_0_ = *χ* ⋅ *p*_0_ and *χ* ≪ 1.

In the next stage (Fig. 7b), the spin rate *ω* is (steeply) increased to Ω_load_ for shrinking the enclosed gas volume to *V*(Ω_load_) < *V*_side_ while *r*_0_(*ω*) = *r*_1_(*ω*) = *r*(*ω*) > *R*_crest_. Then (Fig. 7c), a retention rate Ω_pps_ ≪ Ω_load_ is set so that the enclosed gas expands to *V*(*ω* = Ω_pps_) expands, while *r*_1_(Ω_pps_) stays well below *R*_crest_ to allow for tolerances ΔΩ (11), thus still preventing overflow. At $\omega = {{\Omega }}_{{{{{{\mathrm{pps}}}}}}}^ \ast \,<\, {{\Omega }}_{{{{{{\mathrm{pps}}}}}}}$ (Fig. 7d), the liquid level arrives above the crest channel, i.e., $r_2\left( {{{\Omega }}_{{{{{{\mathrm{pps}}}}}}}^ \ast } \right) \le R_{{{{{{\mathrm{crest}}}}}}}$. Mainly depending on the cross sections *A*_1_, *A*_crest_, and *A*_2_ of the inbound, crest, and outlet sections, respectively, liquid is either transferred into the outer chamber at *R*_cham_ by overflow, or liquid pulley mechanisms.

In more detail, the gas pressure in the side chamber amounts to

17 $$p_{{{{{{\mathrm{side}}}}}}}\left( \omega \right) = \varrho \cdot \bar r_{{{{{{\mathrm{side}}}}}}}{{\Delta }}r_{{{{{{\mathrm{side}}}}}}} \cdot \omega ^2 + p_0 = p_0 \cdot \left( {1 + \chi } \right) \cdot \frac{{V_{{{{{{\mathrm{side}}}}}}}}}{{V\left( \omega \right)}}$$

with the mean value and difference $\bar r_{{{{{{\mathrm{side}}}}}}}$ and Δ*r*_side_ deriving from the liquid levels *r*_0_ and *r*_side_ in the inlet and the side chamber, respectively (Fig. 7). For pneumatic siphon priming to unfold, i.e., to reach Δ*r* > 0, the geometry Γ and liquid volume *U*_0_ have to be configured so

18 $$\begin{array}{l}V\left( {{{\Omega }}_{{{{{{\mathrm{pps}}}}}}}^ \ast } \right) - V\left( {{{\Omega }}_{{{{{{\mathrm{pps}}}}}}}} \right) \ge \left[ {r_1\left( {{{\Omega }}_{{{{{{\mathrm{pps}}}}}}}} \right) - R_{{{{{{\mathrm{crest}}}}}}}} \right] \cdot \left( {A_0 + A_1} \right) + U_{{{{{{\mathrm{crest}}}}}}} + \left[ {r_2\left( {{{\Omega }}_{{{{{{\mathrm{pps}}}}}}}^ \ast } \right) - R_{{{{{{\mathrm{crest}}}}}}}} \right] \cdot A_2\end{array}$$

holds for the gas volume displaced from the side chamber into the main structure while reducing the spin rate *ω* from Ω_pps_ to ${{\Omega }}_{{{{{{\mathrm{pps}}}}}}}^ \ast$.

#### Capillary priming

For priming by capillary pressure *p*_Θ_ (6), the outlet displays a hydrophilic coating to provide a (constant) contact angle $0 \, < \, {{\Theta }} \, \ll \, 90^\circ$ at all interfacial surfaces, and hence *p*_*z*_ = *p*_→_ = *p*_Θ_ > 0. In such a siphon valve (Fig. 6b), the meniscus stops at a first equilibrium position *R*_crest_ < *r*_1_(Ω_cps_) < *R* in the inbound segment of cross section *A*_1_ with a negative offset Δ*r* < 0, i.e., *r*_1_ < *r*_0_. This distribution Λ(Ω_cps_) relates to a retention rate

19 $${{\Omega }}_{{{{{{\mathrm{cps}}}}}}} = \sqrt {\frac{{4\sigma \cos {{\Theta }}}}{{\varrho \cdot \bar r{{\Delta }}r \cdot D}}}$$

(neglecting the small capillary pressure at the meniscus in the large inlet reservoir for *A*_1_/*A*_0_ ≪ 1), which results in Ω_cps_/2*π* ≈ 10 Hz and 16 Hz for water under typical conditions, and Θ = 70° and 0°, respectively; any spin frequency *ω* > Ω_cps_ will retain the liquid.

The second equilibrium position *r*_2_ establishes at $\omega = {{\Omega }}_{{{{{{\mathrm{cps}}}}}}}^ \ast$ with the meniscus at *r*_2_ > *R*_crest_ in the outlet segment of cross section *A*_2_. Any further progression *r* > *r*_2_ of the meniscus for $\omega \,<\, {{\Omega }}_{{{{{{\mathrm{cps}}}}}}}^ \ast$ will then grow Δ*r* to set *p*_*ω*_ + *p*_Θ_ > 0, and thus trigger continuous siphoning. As for the other mechanisms for siphon priming, the choice of the critical rates Ω and Ω* needs to consider their standard deviations ΔΩ and ΔΩ* (11) induced by experimental tolerances {Δ*γ*_*i*_}, and the required reliability quantified by the factor *M*.

As a low-pass valve, capillary-action primed siphons are particularly suitable for LUOs requiring strong centrifugal fields *f*_*ω*_ (1). The spread ΔΩ_cps_ of the threshold frequency Ω_cps_ (12), which might be related to poor definition of the diameter *D* contact angle Θ, is normally of minor practical relevance, as long as Θ stays well below 90°.

In purely capillary-driven priming at *ω* = 0, the time

20 $$T_{{\Theta }} = \frac{{4\eta \cdot l^2}}{{D \cdot \sigma \cos {{\Theta }}}}$$

for covering the axial distance *l* ≈ *L* + *Z* + *L*_crest_ + (*R*_cham_ − *R*_crest_) scales with *l*^2^, the viscosity of the liquid *η*, and inversely with its surface tension *σ*, cos Θ > 0, and the cross-sectional diameter of the (round) channel *D*.

#### Lost volume

**Fig. 8:**
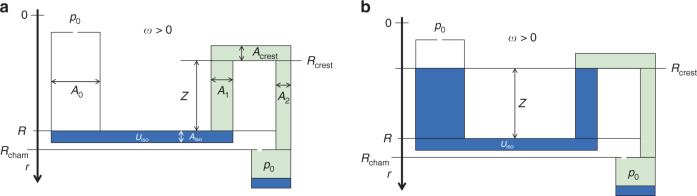
Residual volume in centrifugal siphoning. After the transfer, part of the liquid *U*_0_ is left in the siphon structure. This *U*_loss_ < *U*_0_ sensitively hinges on the dynamics of flow, and the shape of critical parts of Γ, for instance, on the cross sections *A*_0_, *A*_1_, *A*_2_, *A*_iso_, and *A*_crest_ of the radial and isoradial segments; *U*_loss_ would ideally be 0, or at least reproducible, i.e., Δ*U*_loss_ ≈ 0. **a** Liquid $U_{{{{\mathrm{loss}}}}} \approx U_{{{{\mathrm{iso}}}}}$ residing in the siphon structure ${{\Gamma }}$ after “pulley” type siphoning failed to empty the isoradial channel of volume $U_{{{{\mathrm{iso}}}}}$. This volume $U_{{{{\mathrm{loss}}}}}$ detaches from the liquid as air is sucked into the liquid plug. $U_{{{{\mathrm{loss}}}}}$ and its spread ${{\Delta }}U_{{{{\mathrm{loss}}}}}$ may, for instance, be minimized through reduction of $A_1 \cdot Z$. **b** Liquid volume $U_{{{{\mathrm{loss}}}}} = U_{{{{\mathrm{iso}}}}} + \left( {A_0 + A_1} \right) \cdot Z$ remaining in ${{\Gamma }}$ with a purely overflow driven siphoning, which is favored by larger cross sections $A_2$ of the radially outbound channel, for which the liquid just “drizzels” into the outer chamber out after passing the crest point.

Transfer by centrifugal siphoning (Fig. 6) is usually accompanied by a loss *U*_loss_ < *U*_0_ + *U*_Δ_ of the original liquid volume *U*_0_, plus *U*_Δ_ for the case of priming by volume addition (Fig. 8). In “pulley”-type of siphoning, the separation of this residual volume *U*_loss_ occurs when air is drawn into the filled outlet channel during forward pumping, so the initially coherent liquid plug tears apart (Fig. 8a). This residual volume ideally vanishes *U*_loss_/*U*_0_ ≪ 1, or exhibits a small spread Δ*U*_loss_/*U*_loss_ ≪ 1; however, in practice, *U*_loss_ and Δ*U*_loss_ sensitively depend on the hydrodynamic processes and the shape of Γ, and tend to decrease with the cross sections *A*_1_, *A*_crest_, and *A*_2_. Overflow driven liquid transfer running without a pulley mechanism (Fig. 8b) tends to reduce the spread Δ*U*_loss_, while producing larger absolute losses *U*_loss_.

## Centrifugo-pneumatic dissolvable-film siphon valving

**Fig. 9:**
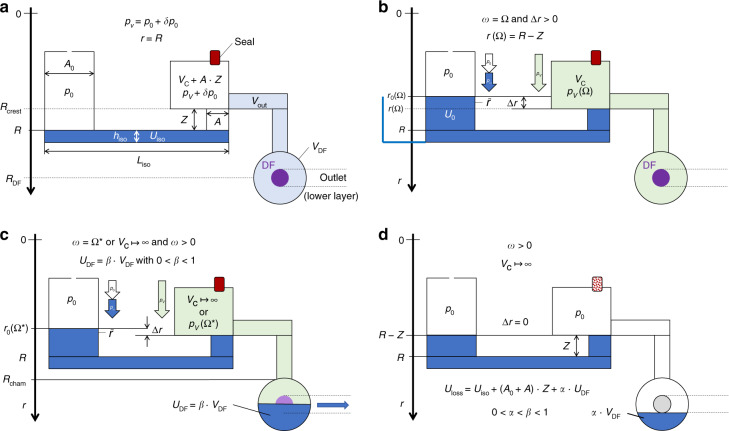
Operational principle of the siphon-shaped CP-DF siphon valve (linearized display, not to scale). The structure Γ features a constant depth *d*. **a** Loading of isoradial segment with $U_{{{{\mathrm{iso}}}}}$ at $\omega = 0$ so a gas volume $V_{{{\mathrm{C}}}} + A \cdot Z$ is enslosed at a pressure $p_0 + \delta p = p_0 \cdot \left( {1 + \chi } \right)$ with respect to the atmosphere at $p_0$ and a small $\chi$ representing potential dynamic effects during priming. **b** Topping up liquid volume to $U_0$ so that the front meniscus settles at $r = R - Z$ when spinning at the retention rate $\omega = \Omega$. **c** At the release frequency ${{\Omega }}^ \ast \,>\, {{\Omega }}$, a sufficient liquid volume $U_{{{{\mathrm{DF}}}}} = \beta \cdot V_{{{{\mathrm{DF}}}}}$ corresponding to a fraction $0 \, < \, \beta \, < \, 1$ of the dead volume $V_{{{{\mathrm{DF}}}}}$ of the outer chamber has overflown to perforate the DF. **d** After the DF membrane has opened at $\omega \,>\, {{\Omega }}^ \ast$, or by venting $V_{{{\mathrm{C}}}} \, \mapsto \, \infty$, and absence of pulley effects, the liquid underneath the crest point $r \ge R - Z$ and $\alpha \cdot V_{{{{\mathrm{DF}}}}}$ with $0 \, < \, \alpha \, < \, \beta$ remains in ${{\Gamma }}$.

The geometry Γ in Fig. 9 constitutes a hybrid of centrifugo-pneumatic (CP) valves (Fig. 3), sacrificial dissolvable-film (DF) barriers (Fig. 5), and centrifugal siphoning (Fig. 6). Its transition between the two hydrostatic equilibrium distributions Λ_*i*∈{1,2}_ results from a centrifugally induced pumping pressure *p*_*ω*_ (3) running against a pneumatic back pressure |*p*_*z*_| = *p*_←_ = *p*_*V*_ (9) from the (initially) sealed receiving chamber. This configuration thus eliminates the need for priming by interim addition *V*_Δ_ (Fig. 6a), hard to manufacture and define circumferential hydrophilic coating Θ < 90° of the narrow outlet channel (Fig. 6b), and difficult to control pneumatic charging of a side chamber (Fig. 7). Liquid transfer merely relies on volume overflow through channel segments exhibiting sufficiently large cross sections *A*.

During retention of this high-pass siphon valve *ω* < Ω, the meniscus stabilizes in the radially inbound section of the siphon channel, thus effectively dampening inertial overshoot propelled by inertia *p*_*m*_ (8) at finite flow rates *Q* > 0, suppressing premature droplet break-off of CP valves (Fig. 3), and radial squeezing of the meniscus for alternative layouts with isoradially directed outlets (Fig. 2, right and Fig. 3, right). Even without direct experimental data, the scheme provides better overall management of loading *U*_0_ with smaller and more reproducible pressure offset *δp*_0_ (and thus ${{\Delta }}\chi \mapsto 0$) than for the basic CP-DF valve (Fig. 3). The gas-tight DF initially isolating the final pneumatic chamber allows for rotationally controlled opening without external actuators, as well as venting mode, while also removing the end-point character of the receiving chamber familiar from basic CP valves (Fig. 3).

By virtue of these manifold synergistical benefits, we consider CP-DF siphon valves as a key enabler for microfluidic large(r)-scale integration (LSI) at high operational reliability, and thus designate a separate section for them. For sake of clarity, we use a simplified geometry Γ (Fig. 9) to represent the valving structures and the resulting, quasi static liquid distributions Λ that lend themselves to a description by closed-form analytical formulas, rather than the previous integrals as, e.g., occurring in (2). The basic concept has been outlined and experimentally validated in a series of prior publications^87,88,112^.

### Functional principle

#### Loading

To best illustrate the basic principle of the CP-DF siphon valving, a somewhat hypothetical, multi-step loading procedure is portrayed in Fig. 9. At rest *ω* ≈ 0, a liquid volume *U*_iso_ completely fills the isoradial section of radial position *R*, length *L,* and height *h*. This way, a pneumatically isolated gas pocket occupies a volume *V*_C_ + *A* · *Z*. The product *A* · *Z* represents the volume of the inbound siphon segment of cross section *A* and length *Z*, while *V*_C_ is mainly composed of the volumes *V*_C,0_ of the large chamber at its inner end, the segmented internal channel *V*_int_, and the final, shallow recess chamber volume *V*_DF_ positioned at *R*_cham_, i.e., typically *V*_int_ + *V*_DF_ ≪ *V*_C,0_ < *V*_C_. The pressure in this gas pocket corresponds to *p*_0_ + *δp*_0_ = *p*_0_ ⋅ (1 + *χ*) with 0 ≤ *χ* ≪ 1, and the ambient pressure *p*_0_ applying to the inlet, which is open to atmosphere, often at *p* ≈ *p*_std_.

The total liquid volume is then topped up to *U*_0_ = *U*_iso_ + *A*_0_ ⋅ [*R* − *r*_0_(Ω)] + *A* ⋅ [*R*−*Z*], so that, at the retention rate *ω* = Ω, the liquid distribution Λ(Ω) places its front meniscus in the inbound segment at *r* = *r*_1_(Ω) = *R*_crest_ = *R*−*Z* (Fig. 9b). For *ω* < Ω, *r* stays in the interval *R* − *Z* < *r*(*ω*) < *R*.

#### Pneumatic pressure

Due to the compression of the enclosed gas volume by *A* ⋅ (*R* − *Z*), the resultant increase in the pneumatic counterpressure

21 $$p_V\left( {R,{{\Gamma }},U_{{{{{{\mathrm{DF}}}}}}},p_0,\chi ,r} \right) = p_ \leftarrow = p_0 \cdot \left( {1 + \chi } \right) \cdot \left( {\frac{{V_{{{{{\mathrm{C}}}}}} + A \cdot Z}}{{V_{{{{{\mathrm{C}}}}}} + A \cdot \left[ {Z - \left( {R - r} \right)} \right] - U_{{{{{{\mathrm{DF}}}}}}}}}} \right)$$

can hence be expressed by *r*, with the liquid volume in the DF chamber *U*_DF_ = 0 vanishing during retention at *ω* ≤ Ω.

#### Meniscus position

Considering that the position of the rear meniscus in the inlet reservoir

22 $$r_0\left[ {R,{{\Gamma }},U_0,U_{{{{{{\mathrm{DF}}}}}}},r\left( \omega \right)} \right] = R - \frac{{U_0 - U_{{{{{{\mathrm{iso}}}}}}} - A \cdot \left[ {R - r\left( \omega \right)} \right] - U_{{{{{{\mathrm{DF}}}}}}}}}{{A_0}}$$

is linear in *r*, the radial product $\bar r{{\Delta }}r$ in (4), and thus also the driving pressure $p_\omega \propto \bar r{{\Delta }}r$ (3), are square functions in *r*. With *p*_→_ = *p*_0_ = const., the hydrostatic equilibrium for the CP-DF siphon valves $p_\omega + p_0 = p_V$ (9) can be written as a cubic function in *r*. Given the algebraic nature of the equation, any advanced symbolic or generic numerical solver can readily produce the results shown.

**Fig. 10:**
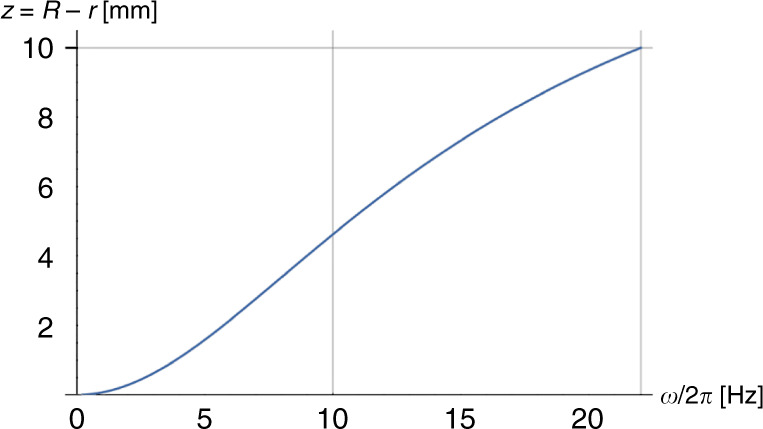
Meniscus position *z* = *R* − *r* as a function of the spin rate *ν* = *ω*/2*π*. With growing *ω*, the meniscus rises in the radially inbound channel until it arrives at *R*_crest_ = *R* − *Z* when $\omega = {{{{\Omega /}}}}2\pi \approx 22\,{{{{{\mathrm{Hz}}}}}}$.

Consequently, algebraic solutions *r* = *r*(*R*, Γ, *U*_0_, *U*_DF_, *p*_0_, *χ*, *ω*) can be found (in principle) for a given geometry of the CP-DF siphon valve Γ, which are parametrized by common experimental parameters, such as the spin rate *ω*, the radial position *R* of Γ, its compression volume *V*_C_ and the loaded liquid volume *U*_0_. Figure 10 displays the rise of the meniscus *z* = *R* − *r* in the inbound segment of the siphon channel until the crest point *R*_crest_ = *R* − *Z* is reached at the critical frequency *ω* = Ω ≈ 22 Hz.

### Liquid retention

#### Critical spin rate

When the front meniscus of Λ assumes *r* = *R*_crest_ at the upper end of the inbound segment (Fig. 9a), inserting *p*_*V*_ (21) into (10) provides

23 $${{\Omega }}\left( {R,{{\Gamma }},U_0,\chi } \right) = \sqrt {\frac{{p_0 \cdot \left[ {\left( {1 + \chi } \right) \cdot \frac{{V_{{{{{\mathrm{C}}}}}} + A \cdot Z}}{{V_{{{{{\mathrm{C}}}}}}}} - 1} \right]}}{{\varrho \cdot \bar r{{\Delta }}r}}} \approx \sqrt {\frac{{p_0}}{{\varrho \cdot \bar r{{\Delta }}r}} \cdot \frac{{A \cdot Z}}{{V_{{{{{\mathrm{C}}}}}}}}}$$

for the critical retention rate Ω of the CP-DF siphon valve.

**Fig. 11:**
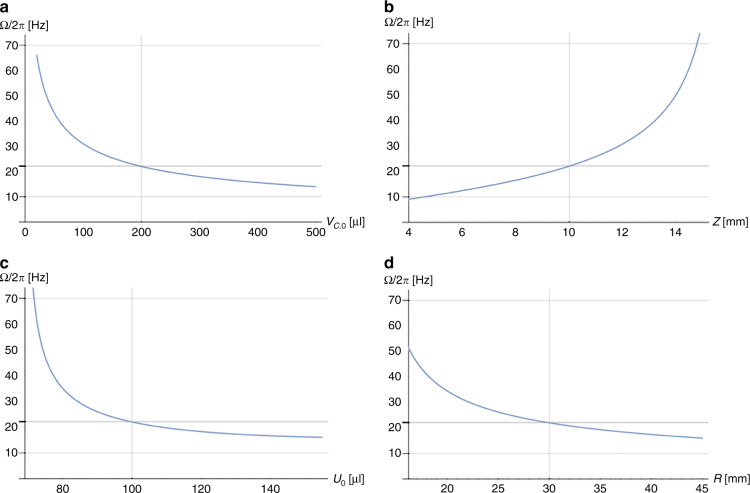
Critical spin frequency Ω/2*π* as a function of individual experimental input parameters (for *χ* ≈ 0). According to (23), Ω depends on (**a**) the compression volume *V*_C_, roughly merging to ${{\Omega }} \propto 1{{{\mathrm{/}}}}\sqrt {V_{{{\mathrm{C}}}}}$. As for a given *U*_0_, Δ*r* rapidly shrinks with growing *Z*, while less affecting $\bar r$, Ω increases steeply towards large *Z* (**b**). For *r*_1_(Ω) = *R* − *Z*, increasing the volume *U*_0_ enlarges Δ*r* faster than $\bar r$ decreases, so the retention rate Ω reduces with *U*_0_ (**c**) and the radial position *R* (**d**). **a** Volume of compression chamber *V*_*C*_. **b** Length of radially inbound sector *Z*. **c** Liquid volume *U*_0_ loaded to Γ. **d** Radial position *R* of Γ.

Figure 11 examines the dependence of the critical spin rate Ω on key experimental parameters. The retention rate Ω is highly configurable, reducing with growing volume *V*_C,0_ of the permanently gas-filled compression chamber (Fig. 11a). Ω also increases by extending the length of the radially inbound segment *Z* (Fig. 11b). As *r*_0_ is linear in *R* and *U*_0_ (22), the radial product $\bar r{{\Delta }}r$ is a square function in *r*_0_, so Ω decreases with *U*_0_ (Fig. 11c) and *R* (Fig. 11d), roughly following 1/*U*_0_ and 1/*R*, respectively.

#### Tuning of the critical spin rate

As *r*_0_(Ω) (22) is linear in *U*_0_/*A*_0_, also the radial product $\bar r{{\Delta }}r$ (4), and thus Ω (23), remain unaltered for *U*_0_/*A*_0_ = const. Hence, an LUO requiring retention of a different liquid volume *U*_0_ preserves the same critical spin rate Ω (23) as long as the cross section of the inlet *A*_0_ is adjusted by the same factor (Γ might also feature a partitioned inlet reservoir in which compartments are flexibly connected by individually configurable barriers).

Figure 11 also reveals that the retention rate Ω (23) may be tuned in the range 10 Hz < Ω/2*π* < 70 Hz. Considering the effort to optimize manufacturing processes to a specific design, it is usually wise to leave the essential, liquid carrying parts of Γ unaltered when adjusting the critical spin rate Ω (23) to the requirements of the assay protocol. Therefore, tuning of Ω (23) is preferentially implemented by the rather large volume of the main compression chamber *V*_C,0_, while preserving the other sectors of Γ. In situations when the radial position *R* needs to be moved, e.g., through spatial requirements, the relation (23) provides a recipe for compensating the shift in *R* by adjusting *V*_C,0_.

Note that the permanently gas-filled sections only contribute with their total (dead) volume *V*_C_ to Ω (23), but they can be partitioned, distributed and located anywhere, as long as being in unfettered pneumatic communication with each other. For instance, the compression volume *V*_C_ might be constituted by a smaller “attachment” to the inner end of the radially inbound section which is connected through a channel of tiny cross section to a larger chamber placed where space would still be available in a multiplexed (disc) layout (see also the advanced geometry displayed in Fig. A1 of Appendix A3).

### Liquid release

#### Modes

Up to now, the considerations have primarily focused on the barrier function of CP-DF valving for 0 < *R* − *r* < *Z* by keeping *ω* < Ω. The opening condition is captured by the overflow of a minimum volume *U*_DF_ = *β* ⋅ *V*_DF_ with 0 < *β* < 1 to sufficiently wet and disintegrate the DF, hence venting the outer chamber of total volume *V*_DF_; Fig. 9c represents the example of *β* = 0.5 for a central location of the DF in a recess of round cross section. After opening the DF at *ω* > Ω* > Ω (Fig. 9, bottom, left), or by perforation of a seal (Fig. 9c), the pneumatic compartment is vented, i.e., $V_{{{{{\mathrm{C}}}}}} \, \mapsto \, \infty$ and $p_V \, \mapsto \, p_0$, and, consequently, any spin rate *ω* > 0 will propel further liquid transfer. On the analogy of Fig. 5, an additional seal, or the DF, might be opened by an external actuator^69,113^, or by a preceding liquid handling step, e.g., through “event-triggering” ^88^.

This transfer of *U*_DF_ = *β* ⋅ *V*_DF_ into the recess reduces the original liquid and gas volumes *U*_0_ and *V*_C_ + *A* ⋅ *Z*, respectively, by *U*_DF_, and typically *U*_DF_ ≪ *U*_0_, while the forward meniscus remains pinned to *r* = *R* − *Z*. We calculate the release rate

24 $${{\Omega }}^ \ast = \sqrt {\frac{1}{{\varrho \cdot \bar r\left( {U_0 - \beta \cdot V_{{{{{{\mathrm{DF}}}}}}}} \right){{\Delta }}r\left( {U_0 - \beta \cdot V_{{{{{{\mathrm{DF}}}}}}}} \right)}} \cdot p_0 \cdot \left[ {\left( {1 + \chi } \right) \cdot \frac{{V_{{{{{\mathrm{C}}}}}} + A \cdot Z}}{{V_{{{{{\mathrm{C}}}}}} - \beta \cdot V_{{{{{{\mathrm{DF}}}}}}}}} - 1} \right]}$$

from (23) by considering the cutback of the loaded volume *U*_0_ upstream of the crest point and the compression volume by *U*_DF_ = *β* ⋅ *V*_DF_ in $\bar r{{\Delta }}r$ (4) and *V* = *V*_C_ − *βV*_DF_ in *p*_*V*_ (5), respectively. These volume reductions lead to a defined increment of the spin rate Ω_step_ = Ω* − Ω, which grows with *U*_DF_. The gap Ω_step_ can thus be tuned for CP-DF siphon valves through Γ, for instance, by the dead volume of the DF chamber outside *r* ≥ *R*_DF_. For common CP-DF siphon valves, *β* ⋅ *V*_DF_/*V*_C_ ≪ 1, so that the 0 < Ω_step_/Ω ≪ 1.

The opening mechanism of the CP-DF siphon valve (Fig. 9) imposes the general volume condition *A*_0_ ⋅ [*R* − *Z* − *r*_0_(Ω)] ≥ *U*_DF_ for both, actuation by rotation or venting, to assure Δ*r* > 0, and thus a non-vanishing centrifugal field *p*_*ω*_ ∝ Δ*r* (3), to drive liquid transfer through the outlet for any *ω >* 0 subsequent to the removal of the DF or seal of the compression chamber. Note that strictly speaking, *ω* < Ω describes “clean” retention without overflow into the DF chamber while, in principle, *ω* < Ω* would be sufficient, as long as {Δ*γ*_*k*_} = 0.

#### Reliability

For consistent CP-DF siphon valving, the forward meniscus needs to stay at *r*(*ω* = Ω − *M* ⋅ ΔΩ) > *R* − *Z* during retention; for reliable rotational actuation, *ω* ≥ Ω* + *M* ⋅ ΔΩ*. Beyond the factors impacting the standard deviation ΔΩ, the uncertainty ΔΩ_step_ is thus mainly determined by the definition of the volume fraction *β* · *V*_DF_. For typical experimental conditions Ω_step_/Ω ≪ 1 and ΔΩ ≈ ΔΩ*, so robust rotational actuation comes down to *ω* ≥ Ω* + *M* ⋅ ΔΩ* ≈ Ω + *M* ⋅ ΔΩ; hence, a “forbidden” frequency band of approximate width 2 · *M* · ΔΩ around *ω* = Ω must be crossed for reliable switching the CP-DF siphon valve (see also Fig. 1).

#### Residual volume

As already investigated in the context of basic siphon valving (Fig. 8), the accuracy and precision of the transferred liquid volume directly enters the mixing ratios underpinning bioanalytical quantitation, and also the Ω (23) and Ω* (24) for subsequent valving steps, and, consequently, critically impacts system-level reliability of microfluidic LSI.

Neglecting inertial and interfacial effects, and assuming purely Δ*r* > 0 driven overflow across the crest channel, and a fraction *α* · *V*_DF_ with 0 < *α* < *β* < 1 remaining in the recess for the DF, the volume

25 $$U_{{{{{{\mathrm{loss}}}}}}} = \left( {A_0 + A} \right) \cdot Z + U_{{{{{{\mathrm{iso}}}}}}} + \alpha \cdot V_{{{{{{\mathrm{DF}}}}}}}$$

constitutes an (approximate) upper boundary of liquid “swallowed” after the transfer (Fig. 9d), with *α* ≈ *β* in common application cases. *U*_loss_ (25) displays a direct contribution of *U*_iso_, and increases linearly with *Z* as well as the cross sections *A*_0_ and *A*. Note that, especially for the here assumed, sufficiently large cross section *A*, “pulley”-type siphoning is largely suppressed, therefore optimizing volume precision by minimizing Δ*U*_loss_; such metering might be further improved via the proper definition of a liquid “cut-off”, e.g., by placing a sharp-edged “liquid knife” within a low dead-volume section.

## Rotational valving schemes

The objective of the digital twin concept presented here is to advise the choice and layout of rotationally controlled valving techniques at the pivot of LoaD systems featuring high functional integration density with “in silico” predictable, system-level reliability for rapid and cost-efficient scale-up of manufacture from prototyping (for initial fluidic testing) to pilot series (for initial bioanalytical testing) and commercial mass fabrication. This section proposes a repertoire of quantitative metrics which guide the selection of the type and layout of rotationally controlled valving for a given scenario. Note that the model underlying the digital twin presented here contains various simplifications, so experimental verification is still needed.

### Performance metrics

#### Critical frequencies and field strengths

For a given high-pass valve, maximum field strengths $f_\omega \left( {{{{\hat{\mathrm \Omega }}}}} \right) \propto {{{\hat{\mathrm \Omega }}}}^2$ (1) for capillary burst (12)

26 $$f_{{\Theta }} \approx \varrho \cdot \bar r \cdot {{\Omega }}_{{\Theta }}^2 \approx \frac{{4\sigma }}{{{{\Delta }}r}}\frac{{\left( { - \cos {{\Theta }}} \right)}}{D}$$

and basic CP valves (16)

27 $$f_{{{{{{\mathrm{CP}}}}}}} \approx \varrho \cdot \bar r \cdot {{\Omega }}^2 \approx \frac{{p_0}}{{{{\Delta }}r}} \cdot \frac{{A \cdot Z}}{{V_{{{{{\mathrm{C}}}}}}}}$$

as well as for the CP-DF siphoning structure (23) of retention rate ${{{\hat{\mathrm \Omega }}}}$ cannot be exceeded during processing of an upstream LUO. For the low-pass mechanisms, there is, per definition, only a critical rate ${{\check{\Omega}}}$ for valve opening at $\omega \, < \, {{\check{\Omega}}}$. In case of the capillary primed siphoning (Fig. 6b), there is a minimum field strength

28 $$f_{{{{{{\mathrm{cps}}}}}}} \approx \varrho \cdot \bar r \cdot {{\Omega }}_{{{{{{\mathrm{cps}}}}}}}^2 \approx \frac{{4\sigma \cos {{\Theta }}}}{{{{\Delta }}r \cdot D}}$$

which will have to be calculated numerically for the pneumatic priming mechanism (Fig. 7). In most LUOs, such a minimum field strength *f* is of minor practical relevance. Note that for particle separation by *f*_*ω*_ (1), $\varrho$ represents the density differential to the suspending medium.

For the CP valves (with *χ* = 0), we find, by revisiting at (16) and (23), that the difference *p*_←_ − *p*_→_ becomes $p_0 \cdot A \cdot Z{{{{{\mathrm{/}}}}}}V_{{{{{\mathrm{C}}}}}}$, so ${{{\hat{\mathrm \Omega }}}} \propto \sqrt {A \cdot Z{{{{{\mathrm{/}}}}}}V_{{{{{\mathrm{C}}}}}}}$. Mathematically, its scaling with $1{{{{{\mathrm{/}}}}}}\sqrt {V_{{{{{\mathrm{C}}}}}}}$ allows raising the retention rate ${{{\hat{\mathrm \Omega }}}}$ to any required value by simply downsizing the compression volume *V*_C_. The same holds for capillary burst valves with *p*_←_ = *p*_Θ_ ∝ *σ* ⋅ cos Θ/*D* (6) with vanishing *p*_→_ ≈ 0 when shrinking the diameter of the constriction *D*. However, in practice, reducing *V*_C_ and *D* is limited by the minimum feature sizes of the manufacturing technologies, and growing spread ΔΩ (11), and also the product *σ* · cos Θ has upper limits for capillary valves.

Apart from its linearity in $\sqrt {p_ \leftarrow - p_ \to }$, we also observe that the retention rate ${{{\hat{\mathrm \Omega }}}} \propto 1{{{{{\mathrm{/}}}}}}\sqrt {\bar r{{\Delta }}r}$ (10) can be increased by minimizing the geometrical product $\bar r{{\Delta }}r$ representing the radial coordinates of the liquid distribution Λ within the valving structure Γ. This dependence unravels a clear advantage of siphoning strategies where the Δ*r* and $\bar r$ only refer to the radial distribution of Λ between the menisci *r*_0_ and *r* in the inlet and inbound section, respectively, while the outer volumes extending between *r* and *R* (plus *U*_iso_) do not enter ${{{\hat{\mathrm \Omega }}}}$ (10), and can thus be sized “randomly”.

Hence, in contrast to the basic capillary (Fig. 2) or CP (Fig. 3) modes where Δ*r = R* + *Z* − *r*_0_ and $\bar r = 0.5 \cdot \left( {R + Z + r_0} \right)$ must hold during retention, siphon valving can be geared for high retention rates ${{{\hat{\mathrm \Omega }}}}$ (10) by “hiding” the bulk liquid volume outside *r*, while minimizing the radial extension Δ*r* or the mean position $\bar r$ of the inner liquid distribution Λ in the radial interval between *r*_0_ and *r*. Note, however, that maximization of ${{\Omega }} \propto 1{{{{{\mathrm{/}}}}}}\sqrt {{{\Delta }}r}$ (23) hits a limit as the volume *A*_0_ · Δ*r* has to be sufficient to effectuate complete filling of the channel section extending between the position *r*_1_ during retention to *r*_2_ for still being able to trigger liquid release.

#### Bandwidth

The metric

29 $$\overline {{{{{\Delta \Omega }}}}} = \frac{{{{{{\Delta \Omega }}}}}}{{\omega _{{{{{{\mathrm{max}}}}}}} - \omega _{{{{{{\mathrm{min}}}}}}}}}$$

reflects the statistical spread of the critical frequency Ω to variations in the experimental input parameters with respect to the practically available spin rate corridor between *ω*_min_ and *ω*_max_. Minimization of $\overline {{{{{\Delta \Omega }}}}}$ (29) can thus guide the development of tolerance-forgiving designs.

For rotationally actuated siphon valving (Fig. 6), the retention and release frequencies Ω and release Ω* can be modified separately. Both spin rates need to be suitably spaced to account for their individual spreads ΔΩ and ΔΩ*; in addition, the differential in the spin rate *ω* needs to allow lifting the meniscus past the second (unstable) equilibrium distribution Λ to *r*_2_ beyond the crest point at *R*_crest_, or even further to deliver a minimum liquid volume *U*_DF_ to the outer chamber for ushering CP-DF siphon valving (Fig. 9). This requires reserving a band ${{\Omega }} - M \cdot {{{{\Delta \Omega }}}} \,<\, \omega \,<\, {{\Omega }}^ \ast + M \cdot {{{{\Delta \Omega }}}}^ \ast$ for the spin rate *ω*. Towards LSI, it is thus favorable to minimize the metric

30 $$\overline {{{{{\Delta \Omega }}}}^ \ast } = \frac{{({{\Omega }}^ \ast + M \cdot {{{{\Delta \Omega }}}}^ \ast ) - \left( {{{\Omega }} - M \cdot {{{{\Delta \Omega }}}}} \right)}}{{\omega _{{{{{{\mathrm{max}}}}}}} - \omega _{{{{{{\mathrm{min}}}}}}}}} \approx \frac{{2 \cdot M \cdot {{{{\Delta \Omega }}}}}}{{\omega _{{{{{{\mathrm{max}}}}}}} - \omega _{{{{{{\mathrm{min}}}}}}}}}$$

in order to be able to “squeeze” as many fluidic operations as possible into the available frequency range.

#### Volume loss

Upon completion of valving, a part *U*_loss_ = ζ · *U*_0_ with 0 ≤ ζ < 1 of the originally loaded volume *U*_0_ might remain in the structure Γ (Fig. 8). While ζ ↦ 0 for the basic valve setups implementing radially directed flow (Figs. 2 and 3), the emptying of the siphon structures (Fig. 6) runs against the centrifugal pressure head *p*_*ω*_ (3) in the inbound section once gas has compromised the integrity of the liquid plug to create a segment characterized by Δ*r* < 0.

As sketched in Fig. 9, such volume loss *U*_loss_ may be approximated by (25) for CP-DF valving. In some bioassays, a systematic loss can be factored in by loading more liquid volume *U*_0_ to the inlet. Still, accommodating *U*_loss_ (25) tends to increase the footprint of liquid handling structures decisively enters the output volume *U*_0_ − *U*_loss_, and thus the frequencies Ω (23) and Ω* (24) of subsequent valving steps in LSI. A metric guiding design optimization might thus be

31 $$\bar U_{{{{{{\mathrm{loss}}}}}}} = \frac{{U_{{{{{{\mathrm{loss}}}}}}}}}{{U_0}}$$

which obviously vanishes when minimizing *U*_loss_ (31).

#### Volume precision

In the same way as the absolute amount of liquid determines the critical frequencies Ω (23) and Ω* (24) of subsequent flow control operations and concentrations in assays, their statistical spreads Δ*U*_0_ and Δ*U*_loss_ impact the precision of the inlet volume *U*_0_ in the next step. While, again, systematic losses may be factored into the valve design Γ and spin protocol *ω*(*t*), stochastic fluctuations may even interrupt liquid handling sequences as the minimum amount of liquid needed to reach Ω* (24) may not be available in the inlets of a subset of valves. We define the dimensionless ratio

32 $$\overline {{{\Delta }}U} _{{{{{{\mathrm{loss}}}}}}} = \frac{{{{\Delta }}U_{{{{{{\mathrm{loss}}}}}}}}}{{U_{{{{{{\mathrm{loss}}}}}}}}}$$

as the metric to be minimized, i.e., $\overline {{{\Delta }}U} _{{{{{{\mathrm{loss}}}}}}}\, \mapsto \, 0$, for enhancing the reliability of multiplexed valving. Alternatively, *U*_loss_ may also be referenced in (32) to *U*_0_.

#### Radial extension

Radial space is precious on centrifugal LoaD systems. To illustrate this, we consider that the centrifugal field *f*_*ω*_ (1) is unidirectional, i.e., it cannot (directly) pump liquids towards the center of rotation; such centripetal pumping would require the provision of power, e.g., connection of a pressure source^100^, chemical reaction^105,110^, imbibition^89,114^, or potential energy in the centrifugal field, e.g., through the simultaneous displacement of a centrally stored (ancillary) liquids^115^ or centrifugo-pneumatic siphoning^67^. Such methods, while technically feasible and successfully demonstrated, would somewhat compromise the conceptual simplicity of the LoaD paradigm.

In purely rotationally controlled LoaD systems considered in this work, the LUOs of (serial) assay protocols are therefore typically aligned in a radially outbound sequence arranged in the order of their execution. This also implies that the reservoirs taking up the sample and reagents to be processed may need to be located centrally. In multi-step assay protocols, the radial confinement of the disc between *R*_min_, e.g., given by the size of an inner hole to clamp the disc to the spindle (*R*_min_ = 75 mm for optical data storage media), plus some space for bonding to a lid, and the largest radius *R*_max_ (in the range of 55 mm for a CD format) at which structures can still be placed, limits the number of LUOs that can be automated. A design goal may therefore be to radially compress each structure of extension Δ*R*_Γ_ of the LUO and its downstream control valve. ${{\Delta }}R_{{\Gamma }} = \hat r - \mathop{{\check{r}}}\limits$ will often correspond to the difference between the minimum radial position of the inner meniscus $\big[\mathop{{\check{r}}}\limits = {{{{{\mathrm{min}}}}}}[r_0\left( \omega \right)]$ over the course of valving *ω*(*t*), and the radially outer edge $\hat r$ of the final receiving chamber. The metric

33 $$\overline {{{\Delta }}R} = \frac{{{{\Delta }}R_{{\Gamma }}}}{{R_{{{{{{\mathrm{max}}}}}}} - R_{{{{{{\mathrm{min}}}}}}}}}$$

might thus be chosen to guide optimization of radial space for a rotationally valved LUO.

#### Real estate

The total area available on the round LoaD device $A_0 = {\int}_{R_{{{{{{\mathrm{min}}}}}}}}^{R_{{{{{{\mathrm{max}}}}}}}} {2\pi \cdot rdr} = \pi \left( {R_{{{{{{\mathrm{max}}}}}}}^2 - R_{{{{{{\mathrm{min}}}}}}}^2} \right)$ is shared between LUOs and their intermittent valves. Therefore, any space savings through the clever design of Γ will enhance the potential for multiplexing. Furthermore, the unidirectional nature of liquid transport implies that the reservoirs taking up the sample and reagents to be processed may need to be located near the axis of rotation.

Overall, these boundary conditions, which are intrinsic to LoaD systems, make central real estate more scarce and thus precious, which we reflect by the metric (“price tag”)

34 $$\bar A = \frac{1}{{R_{{{{{{\mathrm{max}}}}}}} - R_{{{{{{\mathrm{min}}}}}}}}} \cdot \mathop {\int}\limits_{R_{{{{{{\mathrm{min}}}}}}}}^{R_{{{{{{\mathrm{max}}}}}}}} {\frac{{W\left( r \right)}}{{2\pi \cdot r}}} {\mathrm{d}}r$$

where *W*(*r*) represents the total azimuthal width of the valve structure Γ at a radial location *r*, e.g., the length of the isoradial channel *L* in the simplified geometry of the CP-DF siphon valve (Fig. 9). Note that for finite thickness of the fluidic substrate, typically on the order of 1.2 mm for optical storage media derived formats, the area of sectors containing the liquid volume *U*_0_ loaded to the valve cannot be arbitrarily reduced.

#### Valving time

The interval between prompting the opening of a valve and the completion of the liquid transfer through its structure Γ to the subsequent stage involves different processes, which depend on the selected valving mechanism. For the core modes of hydrophobic barriers (Fig. 2) and CP valving (Fig. 3), a transfer time

35 $$T_{{{{{\mathrm{Q}}}}}} \approx \frac{{U_0}}{Q} = \frac{{8\pi \eta }}{{A^2}} \cdot \frac{{U_0 \cdot l}}{{\varrho \cdot \bar r{{\Delta }}r \cdot {{\Omega }}^2}}$$

is obtained for a centrifugally driven flow propelled by a pressure differential $p = p_\omega = \varrho \cdot \bar r{{\Delta }}r \cdot {{\Omega }}^2$ (3) of a liquid of density $\varrho$ and viscosity *η* through the radial outlet of length *l* and cross section *A*.

The approximation (35) neglects start up and exit effects when the channel is only partially filled, and assumes constant $\bar r{{\Delta }}r$ to deliver a stable pumping pressure *p*. However, the product $\bar r\left( t \right){{\Delta }}r\left( t \right)$ changes over the course of liquid transfer. As previously outlined, for siphon valving, $\bar r$ and Δ*r* are calculated from *r*_0_ and *r*, *l* refers to the aggregate axial length of the siphon and outlet channels, and Ω needs to be replaced by the release frequency Ω* for rotational actuation modes.

By assuming typical values *U* = 10 μl, *A* = (100 μm)^2^, *l* = 1 cm, a mean radial position $\bar r = 3\,{{{{{\mathrm{cm}}}}}}$, Δ*r* = 1 cm, Ω = 2*π* ⋅ 25 Hz, and a density $\varrho = 1000\,{{{{{\mathrm{kg}}}}}}\,{{{{{\mathrm{m}}}}}}^{ - 3}$and viscosity *η =* 1 mPa s roughly corresponding to water, we arrive at an order of magnitude for *T*_*Q*_ ≈ 3.4 s (35) for the basic radial valve configurations. When extending *l* by a factor of 5 and reducing Δ*r* by the same factor to account for siphoning, *T*_*Q*_ (35) increases by a factor of 25 to about 1.5 min.

For release mechanisms implementing DFs, the dissolution time *T*_DF_ of the membrane adds to *T*_*Q*_ (35). *T*_DF_ can be set by the formulation and thickness of the film, and may further require a minimum pressure *p*_DF_ on the film located at *R*_DF_ during wetting. Values for *T*_DF_ can range from seconds to minutes, and may display large standard deviation Δ*T*_DF_. Note that various “timing” modules have been developed for LoaD systems, e.g., to delay or synchronize liquid handling time spans required for assay biokinetics^89,111^.

#### Configurability

The previous deliberations and formulas allowing to maintain or tune the retention, burst and release rates Ω = Ω(*R*, Γ, *U*_0_) and Ω*, and their associated bandwidths ΔΩ and ΔΩ* of rotationally controlled valves through the shape and location *R* of Γ and *U*_0_, play an important role for assay automation and parallelization. This digital twin will enable in silico tools offering high predictive power for configuring designs Γ that are optimized for functional integration, reliability and manufacturability^92^. For CP-DF siphon valves (Fig. 9), the far-ranging configurability of the retention rate Ω through the volume of the permanently gas-filled compression chamber *V*_C,0_, which can be located “anywhere” on the disc, and through reduction of Δ*r* by “hiding” liquid volume on the outer part of the structure Γ, provide major benefits (Fig. 11).

Configurability might also be vital regarding other, collective aspects of LSI, such as mechanical balancing of the disc featuring cavities with moving liquid distribution Λ(*t*), minimizing its moment of inertia $I_m = 0.5\pi \cdot \varrho _{{{{{{\mathrm{disc}}}}}}} \cdot R_0^4$, increasing heat transfer, optimization of mold flow for its mass replication, and interfacing that is compatible with standard liquid handling robotics and workflows.

### Comparison

Table 1 compiles select metrics with their typical scaling behavior and value ranges for the previously outlined valving schemes. Note that, due to the plethora of parameters, their wide value ranges, and refined designs, absolute assessments cannot be made; yet the digital twin concept presented here will help choosing and optimizing the valving concept for a given LoaD application.

**Table 1:** Overview of rotationally controlled valving schemes according to common criteria represented by metrics.

Valving principle	Retention	Release	Retention: high Ω	Band width: low ΔΩ	Spatial footprint	Configurability	Low *U*_loss_	Low Δ*U*_loss_	Transfer to next LUO	Manufacturability
Hydrophobic constriction	*ω* ≤ Ω_Θ_ (12)	*ω* > Ω_Θ_	−	−	+	•	+	+	+	−
Centrifugo-pneumatic	*ω* ≤ Ω_cpv_ (14)	Rotational *ω* > Ω_cpv_ *ω* > Ω_cpv_ (16) *U*_DF_ > *β* · *V*_DF_	++	−	+	•	+	+	−	+
		Direct venting *ω* > 0 *V*_C_ ↦ ∞		+		+				−
Siphoning	Δ*r*(*U*_0_) ≤ 0	Volume priming Δ*r*(*U*_0_ + *U*_Δ_) > 0	++	++	•	++	−	++	+	++
	*ω >* Ω_pps_ (18)	Pneumatic priming *ω <* Ω_pps_		−	−	−	•	−		++
	*ω >* Ω_cps_ (19)	Capillary priming *ω <* Ω_cps_		−	•	•	+	−		−
CP-DF siphon valving	*ω <* Ω (23) *U*_DF_ < *β*·*V*_DF_	Rotational *ω >* Ω* (24)	++	+	•	++	−	+	+	•
		Direct venting *V*_C_ ↦ ∞					•			−

Each principle distinguishes by its retention and release mechanism. This benchmarking exercise depends on the particular implementation, so its assessment indicates trends for typical parameters, i.e., *R*, Γ, and *U*_0_, rather than claiming absolute validity for all possible geometries Γ and application scenarios. The rating ranges from very good (++) over good (+) and neutral (•) to unfavorable (−).

## Summary and outlook

### Summary

We have surveyed basic, rotationally controlled valving techniques and modeled their critical spin rates Ω and other performance metrics as a function of their radial positions *R*, geometries Γ and loaded liquid volumes *U*_0_. The underlying digital twin approach allows to efficiently select, configure and optimize the valve towards typical design objectives, such as retention at high field strength during the processing of a Laboratory Unit Operation (LUO) in the inlet reservoir, or to accommodate different reagent volumes *U*_0_.

The modeling presented here specifically correlates retention rates Ω and their standard deviations ΔΩ with experimental input parameters displaying statistical spreads resulting from pipetting, material properties, ambient conditions, and, in particular, the lateral and vertical precision of the manufacturing technique. As a major benefit, this digital twin allows to engineer tolerance-forgiving valve designs displaying predictable functionality along scale-up from prototyping for demonstrating proof-of-concept to pilot series and, eventually, mass manufacture and extended bioanalytical testing. Experimental validation should be implemented once a production technology becomes available that can supply a sufficiently large, and thus statistically relevant number of LoaD devices for thorough characterization on the path to regulatory compliance.

Towards large(r)-scale integration (LSI) of fluidic function underpinning comprehensive sample-to-answer automation of multi-step/multi-reagent bioassay panels, the design-for-manufacture (DfM) capability of the digital twin thus allows maximizing the packing density in real and frequency space while assuring reliability at the system level.

The high predictive power of the in silico approach can thus substantially curb the risk, cost, and time for iterative performance optimization towards high technology readiness levels (TRLs), and thus efficiently supports systematic Failure Mode & Effects Analysis (FMEA), and advancement towards commercialization. The general formalism developed for functional and spatial optimization may well be adopted for other Lab-on-a-Chip platforms and applications.

### Outlook

Several extensions of the rudimentary digital twin approach are proposed, e.g., inclusion of previously introduced flow control by event-triggering, rotational pulsing, and delay modules, or further increase of real estate by vertical stacking of multiple fluidic layers. Valving performance can be improved by the sophistication of layouts^92,93^, e.g., with refined shapes, rounded contours, and anti-counterfeit features^94^, and migration from the hydrostatic model to computational fluidic dynamic (CFD) simulation. An advanced design tool could also include the bioassay kinetics. Moreover, virtual prototyping could be extended by including the simulation of the manufacturing processes of the layouts themselves. This would be particularly suitable for more complex methods like mold flow for injection molding or 3D printing.

Regarding the bigger picture, the ability to create larger-scale integrated, fluidically functional designs with predictable reliability may enable foundry models that are commonplace in mature industries such as microelectronics and micro-electro-mechanical systems (MEMS)^7^. These efforts might be supported by existing initiatives aiming at standardization of interfaces, manufacture, and testing^116–118^. As valving assumes a similar role on centrifugal LoaD platforms as transistors for the emergence of integrated circuits (ICs) in (digital) electronics, the community is well equipped with the presented digital twin approach to develop large(r)-scale integrated “bioCPUs” -Centrifugal Processing Units for implementing multi-step, multi-reagent and multi-analyte bioassay panels.

Follow-up work is already planned on computer-aided, possibly automated optimization of integration density, robustness, and manufacturability. As an open platform concept^119^, the work is meant to encourage honing of design, modeling, simulation, and experimental verification, for instance, within a blockchain-incentivized participatory research model involving crowdsourcing by means of hackathons, citizen science, and fab/maker labs^120–123^. Such community-based organization of research, which are already well-established in the thriving field of blockchain, are particularly attractive for centrifugal microfluidic technologies as key intellectual property (IP), which was mainly filed throughout the 1990s and early 2000s, has now entered the public domain.

## Figures and Tables

**Fig. 12:**
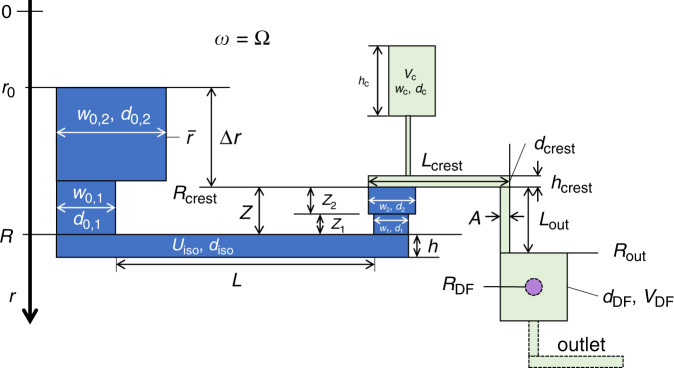
Advanced geometry Γ with partitioned inlet reservoir and inbound segments, each having its individual depth, width, height, and radial position for optimization of key performance metrics (26–35). Note that any permanently gas-filled part of the compression chamber can be randomly shaped and located “anywhere” on the disc (while still pneumatically connected to the liquid Λ).

## Default geometry

The structure Γ, loaded liquid volumes *U*_0_ and radial positions *R* can be varied across a multi-dimensional parameter space, e.g., to tune retention rates Ω, or other key performance indicators. Table 2 gives of overview or generic values which can be used to initiate optimization.

**Table 2:** Default geometrical parameters and relationships of CP-DF siphon valves (Fig. 9).

*R* = 3 cm	*R*_min_ = 1.5 cm	*R*_max_ = 5.5 cm	*R*_DF_ = 3.15 cm > *R*
*A*_0_ = *d*_0_ · *w*_0_	*d*_0_ = 1 mm	*w*_0_ = 5 mm	
*U*_0_ = 100 μl < *A*_0_⋅(*R* − *R*_min_)			
*U*_iso_ = *d*_0_⋅*h*⋅*L* ≪ *U*_0_	*d*_iso_ = 1 mm	*h*_iso_ = 1 mm	*L*_iso_ = 15mm > *w*_0_ + *w*
*U*_*Z*_ = *d* · *w* · *Z*	*d* = 500 μm	*w* = 800 μm ≪ *w*_0_	*Z* = 10 mm
*V*_C,0_ = *d*_C_ ⋅ *w*_C_ ⋅ *h*_C_ ≫ *U*_*Z*_	*d*_C_ = 1 mm	*w*_C_ = 20 mm	*h*_*C*_ = 10 mm
*V*_int_ = *d*_int_⋅*h*_int_⋅*L*_int_ ≪ *V*_C_	*d*_int_ = 200 μm	*h*_int_ = 300 μm	*L*_int_ = 1 cm > 2 *w*
$V_{{{{{{\mathrm{DF}}}}}}} = 0.25\pi \cdot d_{{{{{{\mathrm{DF}}}}}}} \cdot D_{{{{{{\mathrm{DF}}}}}}}^2 \ll V_{{{{{\mathrm{C}}}}}}$	*d*_DF_ = 190 μm	*D*_DF_ = 3 mm	*α* = 0.45, *β* = 0.5

The resulting critical spin rate Ω(*R*,Γ,*U*_0_)/2*π* ≈ 22 Hz. Minimum lateral dimensions are given by the smallest practical diameter of milling head (200 µm). As tools for injection molding are often adopted from optical data storage (e.g., CD, DVD, Blu-ray), a central, 1.5-cm diameter hole and a disc radius of 6 cm with thickness near 1.2 mm, fluidic structures Γ may need to stay within the radial interval between *R*_min_ = 1.5 cm and *R*_max_ = 5.5 cm (plus bonding surface), and an upper limit for the depth of about 1 mm. The minimum depth of cavities is often restricted by the sealing technology; for large lateral extensions or small aspect ratios, sagging of the lid, which is often a foil, may significantly change the nominal volume capacity, also in response to the pressure, and might even lead to sticking to the bottom of the cavity. Economically reasonable mass replication by injection molding typically imposes additional requirements regarding mold flow, e.g., on minimum wall thicknesses *δW*, a rather homogeneous distribution of cavities across the disc, and avoidance of shadowing by structures for central injection.

## Consistency checks

Not all in silico designed structures Γ turn out to provide proper valving with given volumes *U*_0_ and critical spin rates Ω. One reason is manufacturability, the other fluidic function. Table 2 already introduces some rudimentary sanity checks as necessities, but not guaranteeing sufficiency for proper valving. As for other parts of this work, we exclude analysis of biochemical function, which may, for instance, be related to surface adsorption of (bio-)molecules in high surface-to-volume ratio channels, denaturing of biomolecules due to chemical agents leaching from bulk materials, and assembly processes, viability of cells or reaction kinetics.

### Fluidic function

A common design goal is to set a certain retention and release rates Ω and Ω* that are consistent with the valving sequence in frequency space for a liquid volume *U*_0_ prescribed by the assay protocol. At least when discarding manufacturing restrictions, the basic radial layouts Γ displayed in Figs. 2 and 3 may be configured to any given critical frequencies Ω, as long as the inlet reservoir can accommodate *U*_0_, i.e., *U*_0_ < *A*_0_ · (*R* − *R*_min_) for the hydrophobic barrier. Furthermore, the cross section of the outlet *A* needs to be sufficiently small to that surface tension maintains the integrity of the liquid plug.

However, proper functioning of the siphon-type valves (Fig. 6) requires more complex design rules, such as fundamental correlations between radial positions, linear dimensions and volumes are already included for (CP-DF) siphon valves. For example, to allow centrifugally driven outflow, the key radial positions of Γ need to be staggered according to *R*_crest_ < *R* < *R*_out_ for transitioning between the hydrostatic equilibria at *r*_1_ = *r*(*ω*) and *r*_2_ = *r*(Ω*) in the inbound and outbound segments at *ω* = Ω and Ω*, respectively. So *R*_crest_ < *r*_1_ < *R* and *R*_crest_ < *r*_2_ < *R*_out_ needs to hold for the two targetted equilibrium positions of the front menisci. These conditions imply ranges 0 < *U*_0_ − *A*_0_ ⋅ *r*_0_(*R*, Γ, *U*_0_, Ω) − *U*_iso_ ≤ *A* ⋅ (*R* − *R*_crest_) and 0 < *U*_0_ − *A*_0_ ⋅ *r*_0_(*R*, Γ, *U*_0_, Ω*) − *U*_iso_ – *A* ⋅ *Z* − *U*_crest_ ≤ *A* ⋅ (*R*_out_ − *R*_crest_) for the loaded liquid volume *U*_0_. In addition, the priming pressure $p_{{{{{{\mathrm{prime}}}}}}} = p_\omega + p_ \to - p_ \leftarrow$ needs to stay positive along the entire, *ω*-controlled changeover of high-pass (Ω < *ω* < Ω*) and low-pass (Ω* < *ω* < Ω) siphon valves between the two equilibrium positions *r*_1_ = *r*(*R*, Γ, *U*_0_, Ω) to *r*_2_ = *r*(*R*, Γ, *U*_0_, Ω*) of their front meniscus.

### Manufacturability

The choice of the valving technology must also comply with manufacturing restrictions^124,125^ of each scheme availed of during scale-up from prototyping to pilot series production and mass fabrication. While early centrifugal microfluidic platforms were often based on capillary pumping and valving, the local definition of contact angles Θ on all walls including the lid and its stabilization over time under different ambient conditions during storage, transport, and deployment at the end user proves to be challenging. This work, therefore, emphasized valving schemes that would not require a coating step during manufacture.

For each manufacturing technique, there are also technical and economical limitations regarding shapes, aspect ratios, geometrical feature sizes, and their tolerances. For instance, minimum (lateral) dimensions of precision milling (of channels) are imposed by practicable tool diameters, in many cases about 200 μm, but rarely smaller than 100 μm; the tool radius furthermore determines the minimum curvature of (lateral) corners. As milling is a common way to prototype polymer LoaD substrates, and also for patterning replication tools, these restrictions apply to positive and negative, i.e., tool-based structures of the original design. Note also that while milling offers a powerful structuring in a wide range of materials, machine times, tooling cost (and wear) and process development of subsequent replication can go rampant when increasing demands on specifications such as surface quality (on floor and side walls), optical finish, wobble, tool wear other deviations from “native” 2.5- to 3-dimensional geometries.

Especially for common polymer mass replication schemes like injection molding, a minimum wall thickness *δW* between all cavities needs to be enforced. For instance, in the CP-DF siphoning valve in Fig. 9, amongst the distances to be monitored are *Z* > *δW* and *L* = *w*_0_ − *w* > *δW*. Smooth demolding sets upper limits on aspect ratios, and commonly necessitates the inclusion of draft angles, i.e., wall inclinations of the order of 5°–15°. More complex criteria may need to be accounted for, like an even distribution of the hydrodynamic resistance in the tool to avoid shadowing and inhomogeneous solidification during mold flow for the typically central, (compression-)injection of the hot melt. Such collective mechanisms may induce adverse effects, such as wobble and eccentricity of the disc, ridges to compromise pressure-tight bonding, or optical artifacts possibly interfering with detection and customer expectation.

In addition to such “design-for-manufacture” (DfM) considerations of each scheme individually, it also needs to be factored in that the technology along manufacturing scale-up might involve significantly diverging capabilities in (techno-economically) achievable tolerances or shapes; therefore, the design restrictions introduced by the least capable scheme will have to be accounted for to assure seamless scale-up from prototyping to commercial production.

In particular, tool making and optimization of mold flow are decisive cost drivers for microfluidic systems; layouts for new applications, i.e., centrifugally automated assay protocols, should ideally be derived, as much as possible, from designs that have already been previously validated, while only varying parameters that are assumed to be less critical on behalf of fluidics, biology, and manufacturing.

## Advanced design

In Fig. 12, we fine-structure the original geometry for CP-DF siphoning of Fig. 9; each compartment may have its specific depth, width, and height to allow a wider space for multi-parameter optimization according to the performance metrics (26–35). Note that permanently gas-filled parts of the compression volume *V*_C_ may be moved to “any” location available on the disc as long as it is connected by a pneumatic conduit to the DF chamber.

Further variations might include inclination angles with respect to the radial and azimuthal directions, rounded shapes, branched structures to prevent blockage of air flow by residual liquid, liquid knifes for accurate metering of dispensed liquid volumes, low-threshold capillary stops for transient pinning of the meniscus, and draft angles for proper demolding.
